# Life history trade-offs associated with exposure to low maternal capital are different in sons compared to daughters: Evidence from a prospective Brazilian birth cohort

**DOI:** 10.3389/fpubh.2022.914965

**Published:** 2022-09-20

**Authors:** Jonathan C. K. Wells, Tim J. Cole, Mario Cortina-Borja, Rebecca Sear, David A. Leon, Akanksha A. Marphatia, Joseph Murray, Fernando C. Wehrmeister, Paula D. Oliveira, Helen Gonçalves, Isabel O. Oliveira, Ana Maria B. Menezes

**Affiliations:** ^1^Policy, Population and Practice Research and Teaching Department, University College London Great Ormond Street Institute of Child Health, London, United Kingdom; ^2^Faculty of Epidemiology and Population Health, London School of Hygiene and Tropical Medicine, London, United Kingdom; ^3^Department of Community Medicine, UiT the Arctic University of Norway, Tromsø, Norway; ^4^Department of Geography, University of Cambridge, Cambridge, United Kingdom; ^5^Federal University of Pelotas – Postgraduate Program in Epidemiology, Pelotas, Brazil

**Keywords:** maternal investment, life history theory, trade-offs, reproduction, growth, education, inter-generational effect, obesity

## Abstract

**Background:**

Environmental exposures in early life explain variability in many physiological and behavioural traits in adulthood. Recently, we showed that exposure to a composite marker of low maternal capital explained the clustering of adverse behavioural and physical traits in adult daughters in a Brazilian birth cohort. These associations were strongly mediated by whether or not the daughter had reproduced by the age of 18 years. Using evolutionary life history theory, we attributed these associations to trade-offs between competing outcomes, whereby daughters exposed to low maternal capital prioritised investment in reproduction and defence over maintenance and growth. However, little is known about such trade-offs in sons.

**Methods:**

We investigated 2,024 mother–son dyads from the same birth cohort. We combined data on maternal height, body mass index, income, and education into a composite “maternal capital” index. Son outcomes included reproductive status at the age of 18 years, growth trajectory, adult anthropometry, body composition, cardio-metabolic risk, educational attainment, work status, and risky behaviour (smoking, violent crime). We tested whether sons' early reproduction and exposure to low maternal capital were associated with adverse outcomes and whether this accounted for the clustering of adverse outcomes within individuals.

**Results:**

Sons reproducing early were shorter, less educated, and more likely to be earning a salary and showing risky behaviour compared to those not reproducing, but did not differ in foetal growth. Low maternal capital was associated with a greater likelihood of sons' reproducing early, leaving school, and smoking. High maternal capital was positively associated with sons' birth weight, adult size, and staying in school. However, the greater adiposity of high-capital sons was associated with an unhealthier cardio-metabolic profile.

**Conclusion:**

Exposure to low maternal investment is associated with trade-offs between life history functions, helping to explain the clustering of adverse outcomes in sons. The patterns indicated future discounting, with reduced maternal investment associated with early reproduction but less investment in growth, education, or healthy behaviour. However, we also found differences compared to our analyses of daughters, with fewer physical costs associated with early reproduction. Exposure to intergenerational “cycles of disadvantage” has different effects on sons vs. daughters, hence interventions may have sex-specific consequences.

## Introduction

Evolutionary “life history” theory (1, 2) offers a valuable theoretical framework for understanding variability over the life course in health, disease, and human capital. Further advances may be achieved by linking this framework with another conceptual approach that has gained substantial attention during recent decades, the “developmental origins of adult health and disease” (DOHaD) hypothesis (3).

A large volume of research has shown that early-life exposure to adversity (eg poverty, maternal malnutrition, and psychosocial stress) predicts less favourable adult outcomes, and this has now been demonstrated for a wide range of behavioural and physiological traits in numerous populations (4–8). Reducing exposure to early adversity and promoting resilience are thus considered key to decreasing the burden of adult disease and human capital inequalities (9). Some studies have focused on exposure to adverse social experiences within the household during childhood/adolescence (10), however, a range of environmental factors acting during foetal life and infancy also play key roles in these associations (5, 11).

Introducing an evolutionary perspective improves the understanding of *why* such epidemiological associations arise. The key assumption of life history theory is that all organisms are under selective pressure to allocate resources across four specific functions, namely “maintenance”, “growth”, “reproduction”, and “defense”, with trade-offs between them (1, 12, 13). For example, allocating more resources to defence (e.g., the stress response, or immune function) reduces the availability of resources for other functions. Ultimately, natural selection is assumed to prioritise “reproductive fitness” (surviving and reproducing) over maintenance and growth. When fewer resources are available, or when environmental risks are higher, selection should favour increased allocation of resources to immediate survival and reproduction, at a cost to maintenance (a proxy for health) and growth (12, 14). This helps understand why poverty, stress, and malnutrition are so detrimental to health and the development of human capital.

This broad conceptual framework can then be used to help explain why adverse outcomes cluster in specific subgroups of a population. For humans, such clustering was first reported in a New Zealand cohort, where a relatively small segment of the adult population was found to account disproportionately for a range of adverse outcomes, spanning ill-health, risky behaviour, low educational attainment, and criminal convictions (15). This finding was subsequently replicated in larger samples from New Zealand and Denmark (16), however, these studies did not explain why such clustering was occurring.

It is well known that multiple risk factors coalesce around the composite stress of poverty, and their combined effects might potentially explain the clustering of adverse outcomes. For example, poverty is associated with young parental age, maternal malnutrition and infection, parental psychosocial stress, low household income, and lack of parental education (9). These family characteristics may combine with a greater likelihood of other factors outside the household, such as inadequate housing quality, poor public infrastructure, and inadequate access to healthcare and education opportunities. These interactions can be explored using the evolutionary framework we outlined above.

In linking life history theory with the DOHaD hypothesis, however, two issues take on particular importance. First, many of the adverse outcomes can be seen as the product of trade-offs not only between life history functions but also between the present and the future (13, 17, 18). When long-term future benefits appear less attainable, due to higher levels of ecological threat or shorter life expectancy, selection favours “future discounting”, in other words steering resources towards more immediate ends. Second, during foetal life, all ecological stimuli and stresses are transduced through maternal phenotype, and this occurs partially during early postnatal life if the mother is breastfeeding (19, 20), meaning that mothers play a unique role in shaping early trade-offs in their offspring. Third, while early reproduction is not favoured from a policy perspective, it is the primary trait under selective pressure and may indicate an adaptive response to environmental constraints. All of these issues may be particularly relevant to understanding the clustering of adverse outcomes in individuals.

To investigate the developmental origins of clustering of adverse outcomes, we previously analysed data from a Brazilian birth cohort, considering only females in the study (14). We developed a composite score integrating several maternal traits that may shape offspring development (maternal height, BMI, education, and household income). Building on the “embodied capital” model of Kaplan and colleagues (21), all of these traits represent markers of “maternal capital” that underpin the capacity for investment in offspring (14). We showed that low levels of maternal capital shaped life history trade-offs in the daughters that were evident at 18 years of age (14). Specifically, low maternal investment was associated with “future discounting” by the daughter, resulting in investment in traits relevant to defence and reproduction (central fatness, early childbearing) being prioritised over traits that might produce more benefits in the longer term, including better early growth, taller stature, longer education, and less risky behaviour. The results for direct markers of cardio-metabolic health were null, but this might reflect the young age of the cohort (18 years), with low levels of non-communicable disease. Nonetheless, low maternal capital in the offspring's early developmental period already predicted an elevated risk of developing non-communicable disease at later ages, indicated by the clustering of risk markers such as low birth weight, short adult height, higher central fatness and smoking. Because reproduction is expected to reduce the availability of resources for other outcomes, we considered the association of maternal capital with early reproduction especially important in driving trade-offs.

Similar tests have rarely been conducted for sons, indeed substantially less research has been conducted under an evolutionary lens on developmental trajectories in males compared to females, in part, because it is less easy to quantify reproductive outcomes in young adult males (22). Effects of low maternal capital on life history trade-offs might be different for sons compared to daughters, for several reasons. First, the physical investment required for reproduction is different. Whereas female physical investment in reproduction involves the storage of energy in adipose tissue, in order to nourish the offspring during lactation (23, 24), and the metabolic costs and risks of pregnancy, male investment in reproduction is primarily directed towards acquiring “embodied capital” (eg muscle mass, social status), which may promote mating opportunities and paternal investment in the broader sense, rather than the direct provisioning of the offspring (25, 26). As such, while high extrinsic mortality risk in both sexes may be expected to favour early reproduction (27), it is less obvious for males whether this should be traded off against physical growth. Conversely, males on average grow up to be larger than females and have a different anatomical distribution of body fat stores (25), hence there may be costs to health associated with growing and maintaining larger tissue mass in males that are less evident in females. Since males may be subject to weaker trade-offs between growth and reproduction and can delay reproduction to later ages than can women, it is possible that the association between trade-offs and inter-generational stresses might also be different in males, as indicated by previous studies in high-income populations (28–30).

**Figure 1:**
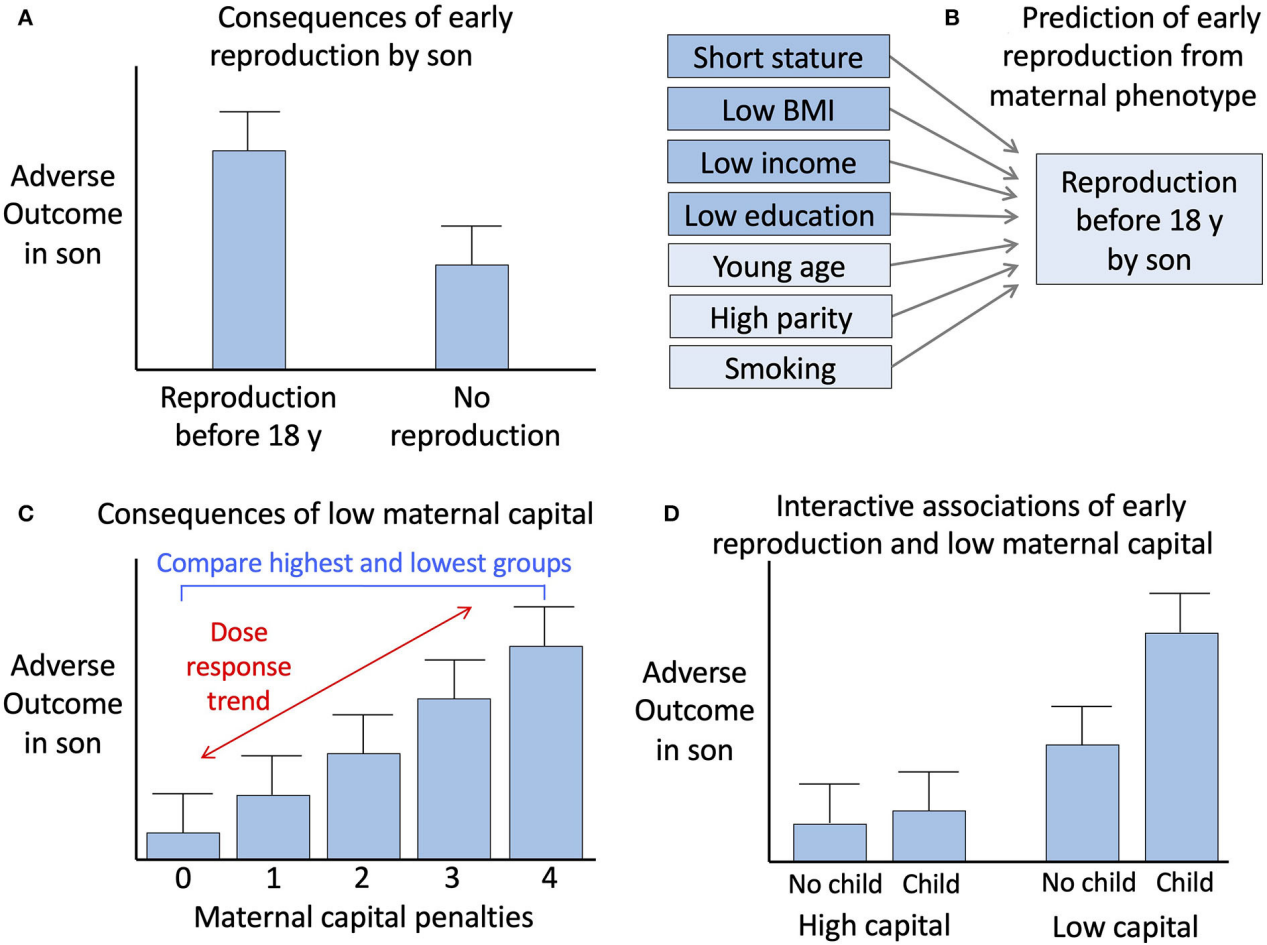
Conceptual diagram of the analysis. **(A)** Comparison of outcomes between sons who reproduced before 18 years vs. those who did not. **(B)** Prediction of early reproduction by sons from components of maternal capital, including those used to generate the maternal capital index and three other maternal traits. **(C)** Analysis of the association of adverse son outcomes with maternal capital, involving assessment of a dose-response trend across the range of maternal capital penalties. **(D)** Analysis of the interactions of early son reproduction and low maternal capital with adverse son outcomes. Reproduced with permission from reference (14).

We therefore undertook in sons the previous analyses we performed for daughters (summarised in Figure 1 and described in detail below).

## Methods

The birth cohort we studied is located in the city of Pelotas (~334,000 inhabitants), in Rio Grande do Sul, the southern state of Brazil. According to data for 1991, shortly before the cohort was established, 91.6% of Pelotas inhabitants lived in urban areas, where the crude birth rate was 19.3 births per 1,000 population, the Human Development Index was 0.558, and the Gini index for income distribution was 0.59 (Atlas of Human Development in Brazil). The profile of the birth cohort itself has been described previously (31, 32) and updated in our previous analysis on the daughters (14).

Offspring birth weight and length were measured at the hospital by the research team. Weight and length at 12 months were measured in a subsample, with an over-representation of those with low birth weight, in the cohort participant's household. At 18 years, weight and height were measured to calculate body mass index (BMI). Waist circumference was measured using a non-elastic measuring tape. Fat-free and fat mass were assessed using air-displacement plethysmography.

Cardiovascular risk markers measured in sons and daughters at 18 years of age follow-up included glucose, glycated haemoglobin (HbA1c), total cholesterol (TC), HDL-cholesterol (HDL-C), LDL-cholesterol (LDL-C), triglycerides (TGL), and systolic (SBP) and diastolic (DBP) blood pressure. The ratio of total cholesterol to HDL was also calculated, with higher values indicating a less favourable cholesterol profile. Venous blood samples were collected regardless of fasting status, left at room temperature for 30min and then centrifuged for 15min at 2,000 g. Serum aliquots were stored at −80°C until analysis. Blood samples were not taken in pregnant or suspected pregnant participants (*n* = 59). Random glucose was measured using an automatic enzymatic colorimetric method. HbA1c was measured using the Variant (Bio-Rad, Hercules, CA) ion-exchange high-performance liquid chromatography (HPLC) method. Lipids were measured using an automatic enzymatic colorimetric method in a biochemistry analyzer (BS-380 Mindray; Shenzhen Mindray Bio-Medical Electronics, China). Systolic and diastolic blood pressures were recorded in the seated position using a calibrated digital wrist monitor (Omron HEM-629, Beijing, China) at the start and end of the visit, with a margin of error of 1 mmHg, and the mean of the two measurements was used in the analysis (33).

A questionnaire was used to ascertain behavioural and human capital outcomes in participants at 18 years of age. Reproductive status was established through the question Have you ever made a girl pregnant? Schooling status was assessed by collecting both categorical data (whether studying now; whether studied in the last year) and continuous data (completed years of education). For those not studying, participants were asked to select from a list of 10 possible reasons accounting for this: difficulty learning; illness; work; no school or travel available; education not considered important; having children; married; violence; failed vestibular examination; other. Participants were asked if they received any income from work, or an allowance (usually from parents) and the amount in Reais (Rs). Questionnaires were also used to establish smoking behaviour (current smoker; had smoked at least 1 cigarette in the last week), and whether the participant had committed a violent crime in the previous year.

### Data processing

We categorised sons according to whether or not they had reproduced early (by 18 years). This age was selected because the follow-up was conducted at this timepoint, and because reproduction before 18 years is widely considered to be early, reflected in the UN definition of early marriage in both sexes as <18 years (34).

We categorised mothers according to their maternal capital at the time the sons were born. This approach combined markers of somatic capital (height and pre-pregnancy BMI) and social or material capital (maternal education and family income) into a composite index. Each of these four traits has been widely associated with phenotypic variability in the next generation (35–38). In Brazil, household income is measured in units of “minimum wages”, where in 1993, 1 minimum wage = US$ 31.4 per month). We treated maternal height as a marker of the mother's own development in early life, maternal BMI as a marker of her current nutritional status, household income as a marker of maternal access to goods and services, and maternal education as a marker of her ability to access beneficial opportunities in society (14).

For each maternal trait, a cut-off was identified defining approximately the lower third in the population, in order to identify those substantially below the median, as described previously (14):

Height: <157 cm
Pre-pregnancy BMI: <21 kg/m^2^
Maternal education: <6 years
Family income: <3 minimum wages

Mothers scored a “capital penalty” for each criterion satisfied, and the penalties were summed to give a total maternal capital score, ranging from 0 to 4 (note that a score of 0 indicates high maternal capital). We used this capital penalty variable to explore continuous associations of maternal capital with son outcomes. For logistic regression analyses, we also divided maternal age into three groups, namely <22 years, 22–28 years, and >28 years. For the same purpose, we divided the sons into three groups in relation to maternal parity, namely first-borns, second-borns, and third-borns or more. However, for descriptive analysis, we also generated a category for high maternal parity, defined as the son being fourth/fifth-born.

To assess the clustering of adverse traits among the sons, we defined three categorical variables at 18 years: obesity as BMI >30 kg/m^2^, short stature as height <171 cm, and school dropout as not studying in secondary school during the previous year. Self-reported current smoking and convictions for violent crime were additional adverse outcomes. Finally, we included low birth weight (defined as <2,500 g) as an adverse outcome, as those with this characteristic remain at elevated risk of non-communicable disease through adult life (39).

### Analytical steps

We undertook several analytical steps, which are summarised in a conceptual diagram (Figure 1). These analyses were identical to those previously undertaken with the cohort daughters, making the results directly comparable.

(A) We first tested whether sons' early reproduction was associated with adverse outcomes, using independent samples *t*-tests or χ^2^ tests. We also examined sons' outcomes in relation to the characteristics of their mothers. We started with this analysis because a key tenet of life history theory is that reduced investment in early life favours the allocation of resources towards early reproduction, at a cost to investment in health and longevity.
(B) To understand how early reproduction by sons was shaped by maternal investment in early life, we tested whether markers of maternal capital predicted sons' reproduction by 18 years. We fitted logistic regression models to test which individual maternal trait predicted the reproductive status of the sons. These models included the four variables defining the composite index of maternal capital, but also additional markers of the capacity for maternal investment (age and parity) as well as risky maternal behaviours (consuming alcohol and smoking during pregnancy) that might constrain maternal investment.
(C) We next fitted regression models to test for dose-response associations of sons' traits with the number of maternal capital penalties. Outcomes included the sons' gestational age and early growth trajectory, breast-feeding experience, adult size and body composition, cardio-metabolic risk markers, risky behaviour (smoking, violent crime) educational attainment, and reproduction status, as well as maternal traits (parity, smoking, alcohol intake).
(D) Finally, we evaluated the extent to which adverse outcomes were clustered among the sons who had reproduced by 18 years of age and also among those who had been exposed to low maternal capital. This was to test whether, as found in daughters, the clustering of adverse traits could be partly attributed to life history trade-offs driven by low maternal capital. We analysed dichotomous outcomes for obesity, short stature, current smoking, school dropout, violent crime, and low birth weight, as justified above. The cut-off for obesity is standard in nutrition research, however, the cut-off for height was selected to define the shortest third of the sample. We also explored the interactions of low maternal capital and sons' early reproduction with these adverse outcomes.

Associations between sons' early reproduction and adverse outcomes might potentially be confounded by direct mother–son transmission of the outcome, due to shared genes or household environments. To exclude this possibility, we fitted regression models (linear or logistic as appropriate) in which associations of the son's early reproduction with outcomes were held constant for the mother's value for the same outcome.

### Statistical analysis

Categorical variables were compared between groups using χ^2^ tests. We tested for normality in the continuous variables, using Q-Q plots and Shapiro–Wilk tests. Not all outcomes were normally distributed, hence we conducted two-group comparisons using both independent *t*-tests and Mann–Whitney U-tests. To adjust for the over-representation of those with low birth weight in the subsample assessed at 1 year, weighted regression was used in the relevant models. Biochemical data were reviewed for potential outliers, resulting in implausibly high values being excluded for glucose (>300, *n* = 3) and triglycerides (>700, *n* = 1). Continuous outcomes reported in the main figures were all natural-log transformed so that subsequent independent samples *t*-tests or Mann–Whitney U-tests express differences (multiplied by 100) in percentage terms (40). Conditional growth was calculated as regression residuals of final size on initial size, divided by the standard error of the estimate (SEE) of the regression model to generate conditional *z*-scores.

To establish which maternal factors independently predicted sons' reproduction status at 18 years, logistic regression models were fitted. Maternal capital variables were divided into two or three groups as follows, in order to identify high-risk groups and test for threshold effects:

Age <22, 22-<28, 28^+^ years
Height <155, 155-<162, 162^+^ cm
Education 0–4, 5–7, 8^+^ years
Income 0–2, 3–4, 5^+^ minimum wages
Parity First-born, second-born, third^+^-born
Smoking in pregnancy Yes, no
Maternal BMI <21, 21–<23.5, 23.5^+^ kg/m^2^

Logistic regression models were also fitted to test the association of the son's phenotype with the odds of early reproduction, without or with adjustment for the equivalent trait in the mother.

To test for associations of low maternal capital and early son reproduction with adverse son outcomes, we conducted χ^2^ tests on dichotomous variables (e.g. being short, out of school etc). We then tested for independent associations of the two predictors, using multiple logistic regression analysis. Due to the small numbers, we defined the low maternal capital group for this analysis as having 3 or 4 capital penalties and compared it against those with no capital penalties. As our previous analysis of this issue using a different method (comparing χ^2^ tests using the likelihood ratio), we also ran these models for the sons. Four individuals had high outlying values for both HbA1c and blood glucose, but the group comparisons were unaffected by whether these individuals were excluded or not.

All analyses were conducted in SPSS version 24 (IBM Corporation, Armonk, New York) and R version 3.6.3 (The R foundation for Statistical Computing, Vienna).

## Results

Of the 2,603 male participants included at birth, 2,024 (78%) participated in the follow-up at the age of 18 years and provided questionnaire responses, of whom 1,970 were measured for anthropometry and body composition, 1,988 for blood pressure, and 1,933 had a blood test. Compared to those followed up, those not followed up had lower birth weight (−135 g, 95%CI −27, −188), birth length (−0.3 cm, 95%CI −0.5, −0.0), gestational age (−0.2 weeks, 95%CI −0.3, −0.0), and maternal pre-pregnancy BMI (−0.5 kg/m^2^, 95%CI −0.9, −0.2). Though statistically significant, these differences were all small, and there were no other differences in baseline maternal or child characteristics (Supplementary Table 1). Likewise, among those followed up, there was little missing data, and those missing data showed either small differences or no differences, in background characteristics, compared to those available for analysis (Supplementary material). The subsample visited at 1 year comprised 652 boys.

### Life-history trade-offs in association with early reproduction of sons

**Figure 2:**
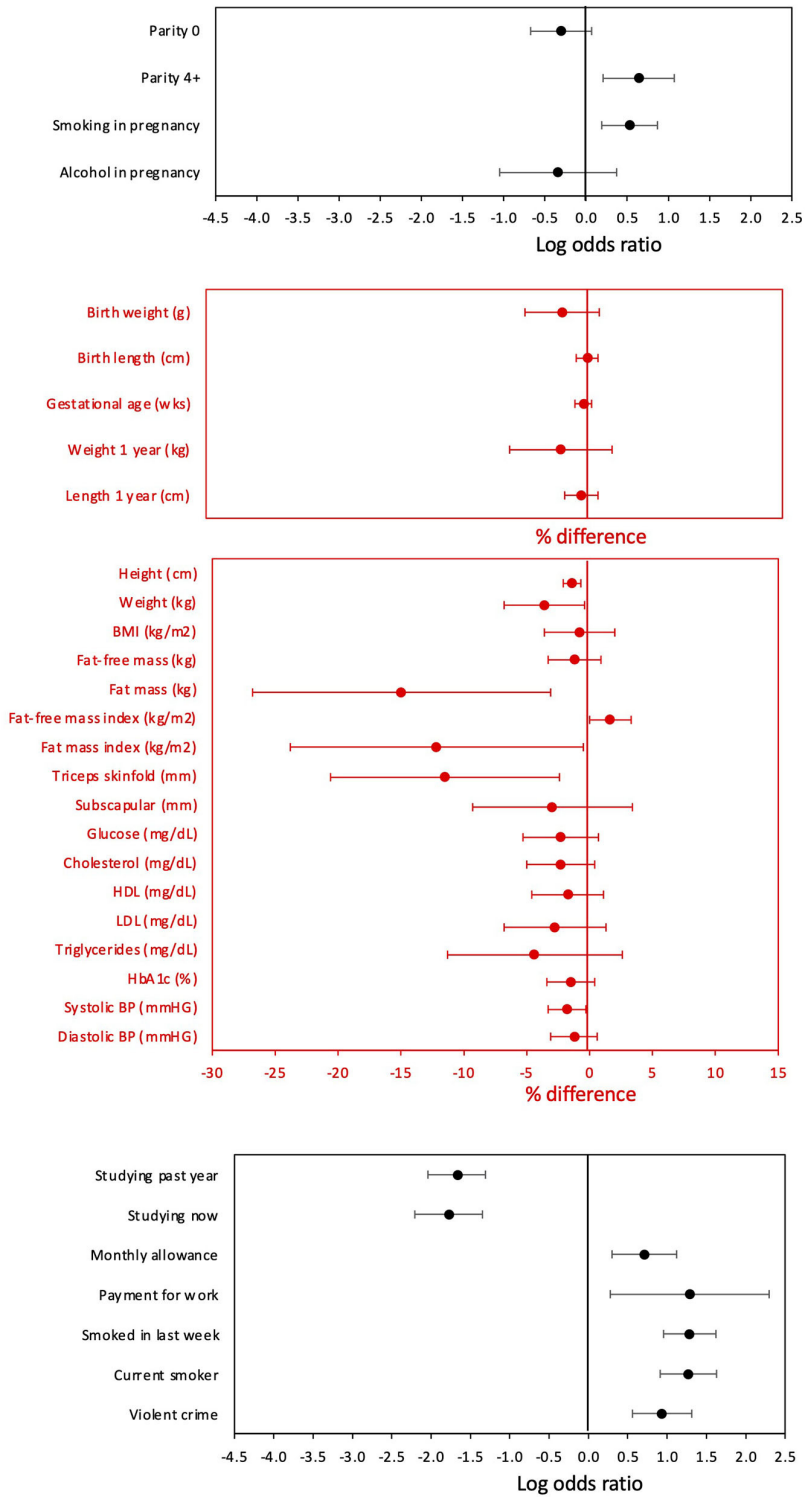
Differences in maternal and son traits from pregnancy to adulthood between sons with or without offspring by 18 years. Categorical variables are shown as odds ratios on a log-scale and 95% confidence intervals. Continuous variables are shown as percent differences and 95% confidence intervals, calculated from natural log-transformed variables. Numerical values for all comparisons are given in Supplementary Table 2.

**Figure 3:**
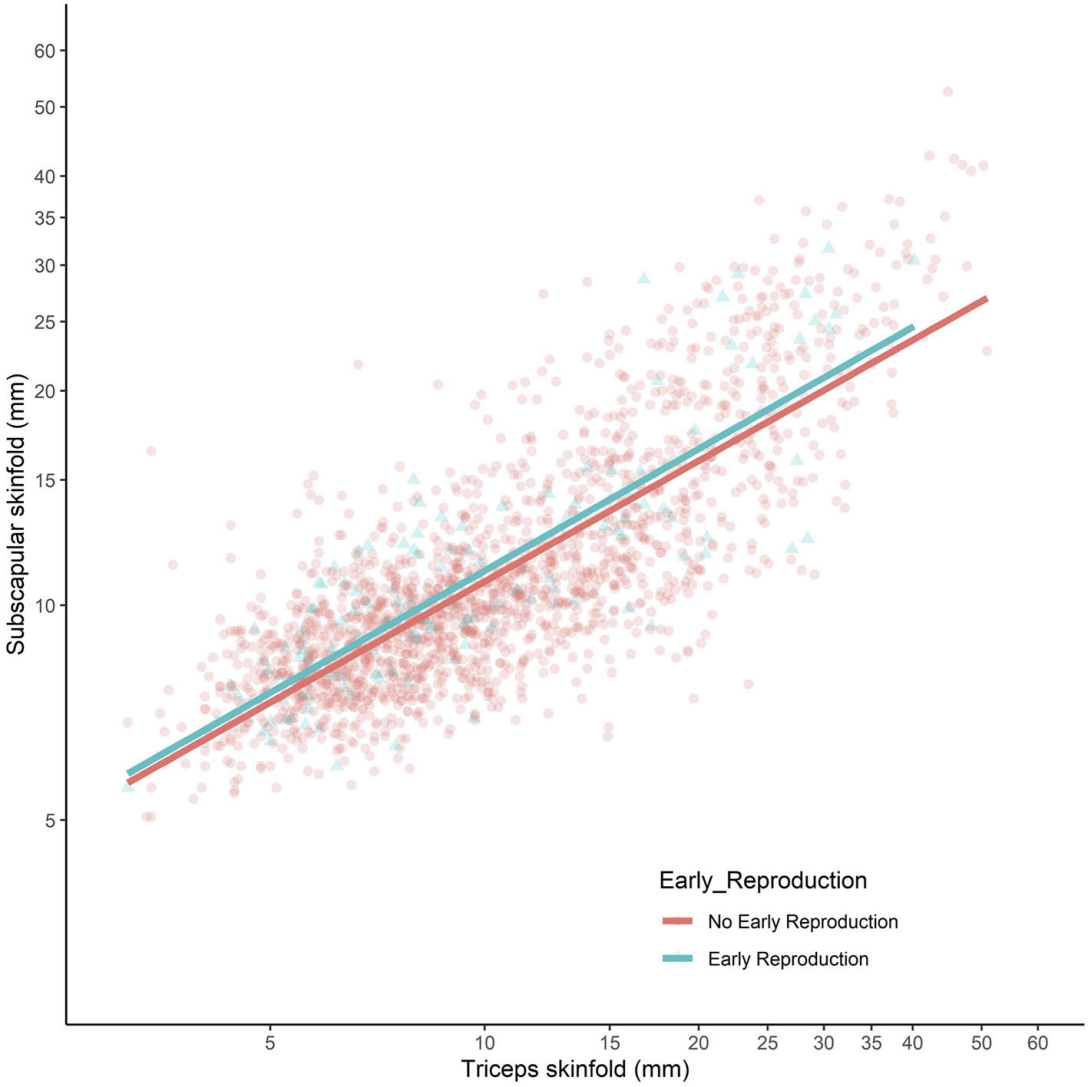
Subscapular skinfold plotted against triceps skinfold in the adult sons, stratified by whether they had reproduced by 18 years (blue scatter and line) or not (red scatter and line). Both axes present log-scales. Early reproducing sons did not differ in their subscapular skinfold (Δ = 0.4 mm, 95% CI −0.2, 0.9) for a given triceps skinfold, indicating no difference in central fat deposition.

The comparison of sons who had or had not reproduced by 18 years is summarised in Figure 2. The results were near-identical, whether independent samples *t*-tests or Mann–Whitney U-tests were used (Supplementary Table 2). Sons who had reproduced by 18 years (*n* = 150) were shorter (−2.5 cm, 95%CI −3.5, −1.4), lighter in weight (−2.7 kg, 95%CI −5.1, −0.3), and had lower fat mass (−2.0 kg, 95%CI −3.4, −0.7) and triceps skinfold (−0.7 mm, 95%CI −1.9, 0.4) compared to those who had not reproduced. However, they did not differ in fat-free mass or fat distribution, as measured by the residual of subscapular skinfold adjusted for triceps skinfold (Figure 3). These patterns indicated that constraints on linear growth and fat deposition are associated with early reproduction.

**Figure 4:**
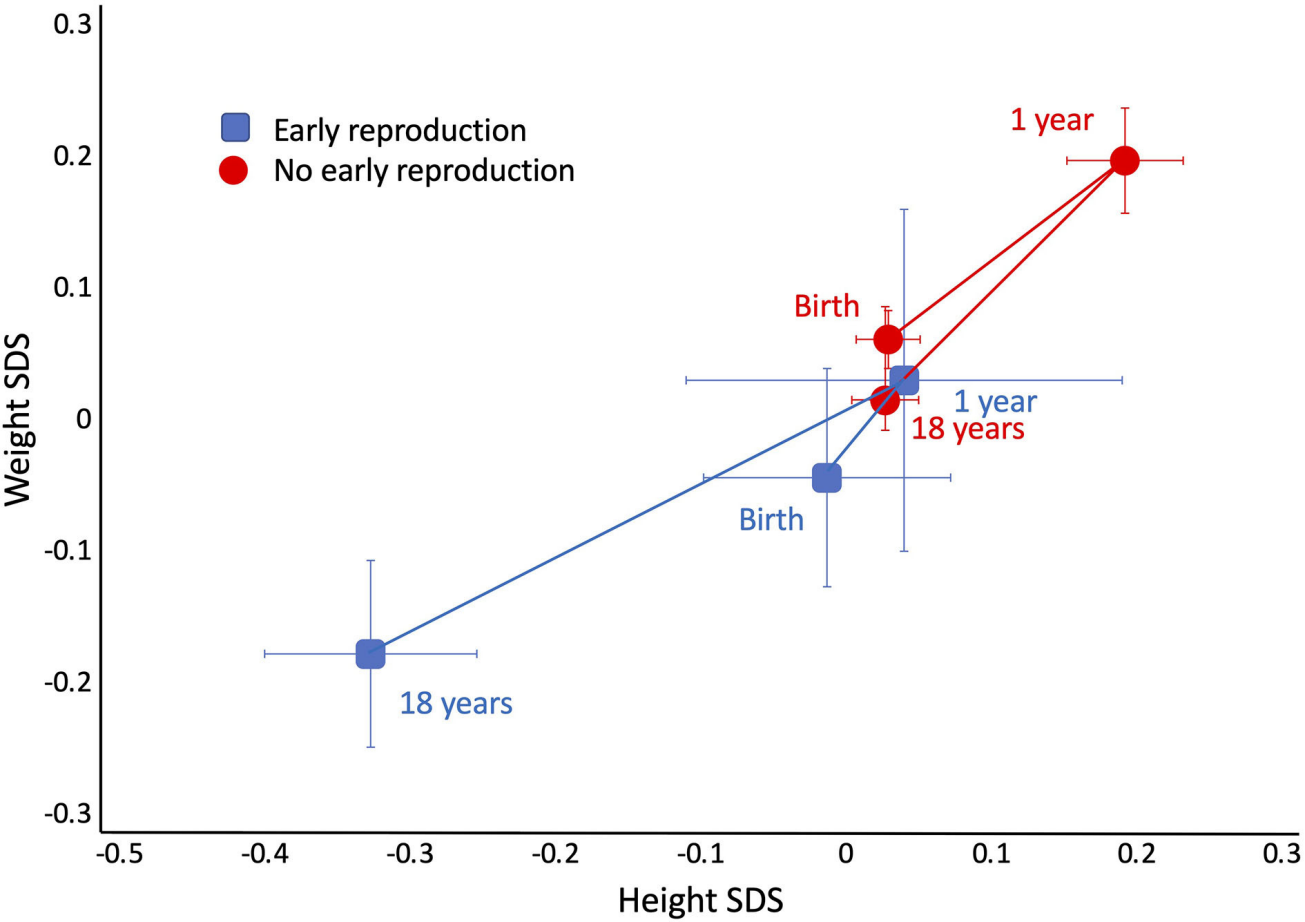
Trajectories of growth in length/height *z*-score and weight *z*-score in the sons, stratified by whether they had reproduced by 18 years (blue points) or not (red points). Early reproducing sons were similar to their non-reproducing sons at birth, and both groups showed a small degree of catch up between birth and 1 year. From this time point, early reproducing sons showed poorer growth, resulting in lower weight and height at 18 years.

Despite these differences in adult size, there were no differences in size at birth or in infancy. Figure 4 contrasts the growth trajectories of the two groups, using data on weight and height z-score at birth, 1 year and 18 years. Neither group differed from the growth reference or each other at birth and 1 year. However, from 1 year, the early reproducing group showed poorer growth in both weight and height, reflected in significantly lower z-scores at 18 years of age. After adjusting for maternal capital, the deficit in the son's stature associated with early reproduction (−2.5 cm, 95%CI −3.6, −1.3) was reduced by 1 cm but was still significant (−1.4 cm, 95%CI −2.5, −0.2), implicating a direct association of the son's own growth trajectory with early reproduction, independent of maternal size. Sons who reproduced early had lower total cholesterol and lower systolic BP, but no other differences in cardiovascular risk.

Regarding their behaviour, early-reproducing sons were more likely to be a current smokers (OR 3.6, 95%CI 2.5, 5.1) and to be receiving payment for work (OR 3.6, 95%CI 1.3, 10), less likely to have been studying in the last year (OR 0.19, 95%CI 0.13, 0.27), and more likely to have committed violent crime (OR 2.5, 95%CI 1.7, 3.7). Among a subset of those out of school (*n* = 420), the most common reason for not being in school for both groups was work – this was slightly more common among early-reproducing than non-reproducing sons (62% and 56%, respectively) (Table 1). Beyond that, 18% of non-reproducing sons did not consider education important, compared to only 10% of the early-reproducing sons. Conversely, 10% of the early-reproducing sons listed “having children” as the main reason for not being in school. When those giving this response were omitted from the analysis, as it was only relevant to one group, there was no difference between the groups in the reason for leaving school.

**Table 1:** Reasons for sons not studying (*n* = 420), stratified by their reproduction status at 18 years.

	**Has reproduced**		**Has not**	
	**(*****n =*** **81)**		**reproduced**	
			**(*****n =*** **339)**	
**Reason for not studying^a^^,^^b^**	***N***	**%**	***N***	**%**
Difficulty learning	4	5	16	5
Illness	0	0	9	3
Work	50	62	189	56
No school or travel available	1	1	9	3
Education not considered important	8	10	61	18
Having children	8	10	0	0
Married	1	1	0	0
Violence	0	0	6	2
Failed vestibular examination	0	0	11	3
Other	9	11	38	11

a Cohort subsample with detailed data on education status.

b Group difference p < 0.0001 if “having children” included as a response, but p = 0.076 if this option excluded.

Sons who had reproduced by 18 years of age were more likely to have been born to high parity mothers (OR 1.9, 95%CI 1.2, 2.9). Their mother was more likely to have smoked in pregnancy (OR 1.7, 95%CI 1.2, 2.4), but was no more likely to have drunk alcohol (OR 0.7, 95%CI 0.3, 1.4).

Using logistic regression analysis, however, the only maternal traits that independently predicted early reproduction by the son were lower/middle levels of maternal education, and smoking during pregnancy (Table 2).

**Table 2:** Logistic regression of son reproducing by 18 years on components of maternal capital (*n* = 1,922).

**Maternal capital**	**Nagelkerke's**		
**component**	***r*^2^ = 0.16**		
	**Odds ratio**	**95% CI**	***p*-value**
**Age**			
<22 years	1.2	0.7, 2.0	0.4
22–28 years	1.0	0.6, 1.6	0.9
28+ years (reference)	1	-	-
**Height**			
<155 cm	1.1	0.7, 1.7	0.6
155–162 cm	0.8	0.6, 1.3	0.3
162+ cm (reference)		-	-
Education	1		
0–4 years	2.6	1.5, 4.3	0.001
5–7 years	2.1	1.3, 3.5	0.004
8+ years (reference)	1	-	-
**Income**			
0–2 minimum wages	1.4	0.8, 2.4	0.2
3–4 minimum wages	0.7	0.4, 1.4	0.3
5+ minimum wages (reference)	1	-	-
**Parity**			
First-born (reference)	1	-	-
Second-born	0.9	0.6, 1.5	0.7
Third-born or higher	1.4	0.8, 2.33	0.2
**Smoking in pregnancy**			
No (reference)	1	-	-
Yes	1.4	1.0, 2.1	0.045
**Maternal BMI**			
<20 kg/m^2^	1.2	0.8, 2.0	0.3
20–23.49 kg/m^2^	1.3	0.9, 2.0	0.2
23.5+ kg/m^2^ (reference)	1	-	-

Grey shading indicates association significant at p < 0.05.

### Associations of maternal capital with son traits

The maternal capital score is the sum of penalties across the four traits, ranging from 0 to 4. The characteristics of individual maternal traits varied in a dose-response manner in association with the composite score (Supplementary Table 3). Thus, as expected, the fewer the penalties, the greater the mother's height, BMI, income, and education. The composite index also showed an inverse dose-response association with maternal age, but a positive association with the frequency of high parity. Thus, low capital mothers were younger and of higher parity, and their sons had on average to compete with more siblings for maternal investment.

**Figure 5:**
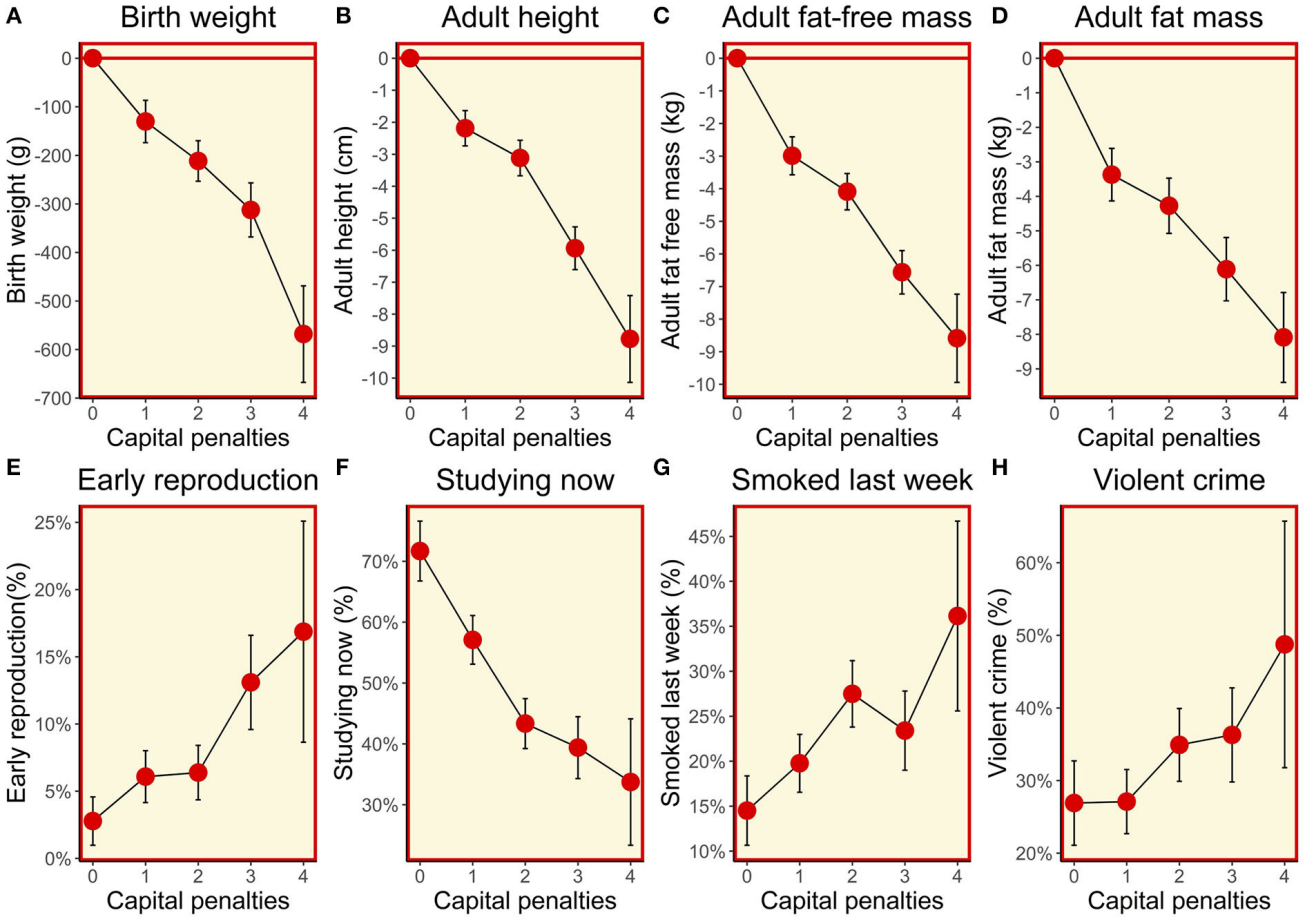
**(A–H)** Dose-response associations between sons' traits and the magnitude of maternal capital, categorised in terms of a total composite score of “penalties” (ranging from 0 to 4) selected from the categories “short stature”, “low body mass index”, “low education” and “low family income”. See text for details of how the index and its categories are defined.

The composite maternal capital index showed a dose response association with the vast majority of outcomes in sons. The lower the level of maternal capital, the higher the proportion of sons that had reproduced early, were out of school, and were current smokers; however, there was no association with the proportion who were being paid an allowance or for work, or who had committed a violent crime (Table 3; Figure 5).

**Table 3:** Dose response associations of maternal or sons' traits according to the number of penalties in maternal capital.

**Predictor**	**0 penalties (*****n =*** **389)**		**1 penalty (*****n =*** **617)**		**2 penalties (*****n =*** **573)**		**3 penalties (*****n =*** **299)**		**4 penalties (*****n =*** **69)**		
**Maternal Traits**	***N***	**%**	***N***	**%**	***N***	**%**	***N***	**%**	***N***	**%**	***p*–value^a^**
First–born	132	40.4	233	39.2	195	34.5	108	29.9	27	32.5	0.017
Fourth^+_^born	20	6.1	47	7.9	83	14.7	59	16.3	9	10.8	<0.0001
Maternal smoking	73	22.3	161	27.1	191	33.7	157	43.5	36	43.4	<0.0001
Maternal alcohol	15	4.6	29	4.9	20	3.5	19	5.3	5	6.0	0.6
	**Coeff**	**95%CI**	**Coeff**	**95%CI**	**Coeff**	**95%CI**	**Coeff**	**95%CI**	**Coeff**	**95%CI**	
Maternal age (y)	28.0	27.3, 28.7	−1.7	−2.6, −0.8	−2.3	−3.2, −1.4	−2.8	−3.8, −1.9	−4.8	−6.4, −3.3	<0.0001
	**Constant**		**1 penalty** ^$^		**2 penalties** ^$^		**3 penalties** ^$^		**4 penalties** ^$^		***p*** **–value** ^b^
**Son Traits**	**Coeff**	**95%CI**	**Coeff**	**95%CI**	**Coeff**	**95%CI**	**Coeff**	**95%CI**	**Coeff**	**95%CI**	
Birth weight (g)	3,441	3,385, 3,497	−134	−204, −64	−211	−282, −141	−311	−388, −233	−569	−64, −445	<0.0001
Birth length (cm)	49.7	49.5, 50.0	−0.3	−0.6, −0.0	−0.5	−0.8, −0.2	−1.0	−1.4, −0.7	−1.9	−2.4 −1.3	<0.0001
Gestational age (w)	39.0	38.8, 39.2	−0.3	−0.5, 0.1	−0.3	−0.5, 0.1	−0.4	−0.6, −0.2	−0.6	−0.9, −0.2	<0.0001
Excl. breastfed (d)^*^	29.7	23.5, 35.8	−8.7	−16.2, −1.2	−16.5	−24.1, −9.0	−17.5	−26.0, −9.0	−17.8	−30.9, −4.8	<0.0001
Weight 1 year (kg)^*^	11.0	10.7, 11.2	−0.6	−0.9, −0.2	−1.0	−1.3, −0.7	−1.2	−1.6, −0.9	−1.8	−2.4, −1.2	<0.0001
Length 1 year (cm)^*^	77.4	76.8, 78.0	−1.6	−2.4, −0.8	−2.3	−3.1, −1.6	−3.2	–−4.1, −2.3	−4.6	−6.0, −3.2	<0.0001
Height (cm)	177.0	176.2, 177.7	−2.2	−3.1, −1.3	−3.1	−4.0, −2.2	−5.9	−6.9, −4.9	−8.8	−10.4, −7.2	<0.0001
Weight (kg)	78.3	76.8, 79.8	−6.4	−8.2, −4.5	−8.4	–−10.3, −6.5	−12.7	−14.7, −10.6	−16.7	−20.0, −13.3	<0.0001
BMI (kg/m^2^)	24.9	24.5, 25.4	−1.4	−2.0, −0.9	−1.8	−2.4, −1.2	−2.5	−3.2, −1.9	−3.2	−4.2, −2.2	0.020
Triceps (mm)	14.7	13.9, 15.6	−2.2	−3.2, −1.2	−3.0	−4.0, −1.9	−4.7	5.8, −3.5	−6.1	−7.9, −4.3	<0.0001
Subscapular (mm)	13.8	13.2, 14.4	−1.7	−2.5, −1.0	−2.0	−2.7, −1.2	−3.2	−4.1, −2.4	−4.0	−5.3, −2.6	<0.0001
Fat–free mass (kg)	61.7	60.9, 62.4	−3.0	−3.9, −2.0	−4.1	−5.0, −3.1	−6.6	−7.6, −5.5	−8.6	−10.3, −6.9	<0.0001
Fat mass (kg)	16.6	15.6, 17.7	−3.4	−4.7, −2.1	−4.3	−5.6, −3.0	−6.1	−7.5, −4.7	−8.1	−10.4, −5.8	<0.0001
Glucose (mg/dL)	93.2	91.4, 94.9	−0.4	−2.6, 1.8	−0.4	−2.6, 1.8	0.9	−1.5, 3.3	2.1	−1.7, 5.9	0.6
Cholesterol (mg/dL)	159.0	156.3, 161.7	−5.4	−8.7, −2.0	−7.9	−11.2, −4.5	−9.5	−13.2, −5.8	−10.0	−15.9, −4.1	<0.0001
HDL (mg/dL)	52.9	51.9, 53.9	−0.8	−2.1, 0.4	−1.4	−2.6, −0.2	−1.4	−2.8, −0.1	−3.5	−5.6, −1.4	0.001
LDL (mg/dL)	88.1	85.9, 90.4	−3.4	−6.2, −0.7	−4.7	−7.5, −1.9	−6.4	−9.4, −3.3	−5.2	−10.1, −0.4	<0.0001
HDL/Cholesterol ratio	0.64	0.61, 0.66	0.01	−0.01, 0.04	0.02	−0.01, 0.04	0.02	−0.01, −0.05	−0.02	−0.02, 0.02	0.6
Triglycerides (mg/dL)	87.7	82.7, 92.7	−4.8	−11.0, 1.5	−8.8	−15.1, −2.5	−11.4	−18.3, −4.5	−11.5	−22.5, −0.6	<0.0001
HbA1c (%)^¥^	4.97	4.91, 5.03	0.03	−0.05, 0.11	0.00	−0.08, 0.08	−0.04	−0.12, 0.05	−0.05	−0.2, 0.08	0.1
Systolic BP (mmHg)	132.6	131.3, 133.9	−2.0	−3.6, −0.3	−1.4	−3.0, 0.3	−3.9	−5.7, −2.1	−4.9	−7.7, −2.0	<0.0001
Diastolic BP (mmHg)	71.7	70.8, 72.5	−0.8	−1.9, 0.2	−0.1	−1.2, 1.0	−1.6	−2.8, −0.4	−1.9	−3.8, 0.1	0.027
Education (y)	9.7	9.5, 10.0	−0.9	−1.2, −0.6	−2.1	−2.4, −1.8	−2.4	−2.8, −2.1	−3.2	−3.7, −2.7	<0.0001
Monthly allowance (Rs)	436	355, 516	120	19, 221	125	24, 226	121	9, 233	195	11, 378	0.025
Gross monthly pay (Rs)	542	476, 609	−43	−38, 123	85	5, 165	44	−42, 130	83	−49, 215	0.1
	**0 penalties**		**1 penalty**		**2 penalties**		**3 penalties**		**4 penalties**		
	***N***	**%**	***N***	**%**	***N***	**%**	***N***	**%**	***N***	**%**	***p*** **–value** ^a^
Reproduction by 18 y	9	2.8	36	6.1	36	6.4	47	13.1	14	16.9	<0.0001
Studying past year	302	92.9	491	82.9	428	75.9	262	73.0	56	67.5	<0.0001
Studying now	233	71.7	338	57.1	244	43.3	141	39.3	28	33.7	<0.0001
Allowance last month	223	68.6	390	65.9	389	69.0	234	65.2	52	62.7	0.5
Pay last year	205	88.7	434	90.8	458	91.2	301	93.2	68	90.7	0.4
Smoked last week	47	14.5	117	19.8	155	27.5	84	23.4	30	36.1	<0.0001
Current smoker	21	6.5	81	13.7	105	18.6	64	17.8	20	24.1	<0.0001
Violent crime	62	22.0	105	19.9	123	24.6	77	24.4	13	19.4	0.3

p-value^a^ chi-square test; p-value^b^ test for trend across the maternal capital groups, by regressing each outcome on a single variable coded 0–4 capital penalties.

* subsample, with analysis adjusted for over-recruitment of low birth weight infants.

$ Each outcome was regressed on four dummy variables, whereby the son's mother was identified as having 1, 2, 3, or 4 capital penalties (0 penalties = reference group).

The mean coefficient and its 95% CI intervals are shown for each dummy variable. Penalties refer to maternal short stature, low BMI, low education or low family income.

¥ HbA1c shown as percentage of total haemoglobin.

n = 2,024, small level of missing data for body composition and cardio-metabolic outcomes as described in text.

Grey shading indicates association significant at p < 0.05.

Maternal capital was positively associated in a dose-response manner with the son's adult size, and fat and fat-free components of adult body composition. These associations had their origins in foetal life, as similar associations were evident for size at birth and during infancy (Figure 5). In all cases, the higher the level of maternal capital, the greater the magnitude of the growth trait or tissue mass in the son. Gestational age was also greater among sons of higher-capital mothers, and the duration of exclusive breastfeeding was longer (test for trend *p* < 0.004), though the magnitude of the effect was small.

**Figure 6:**
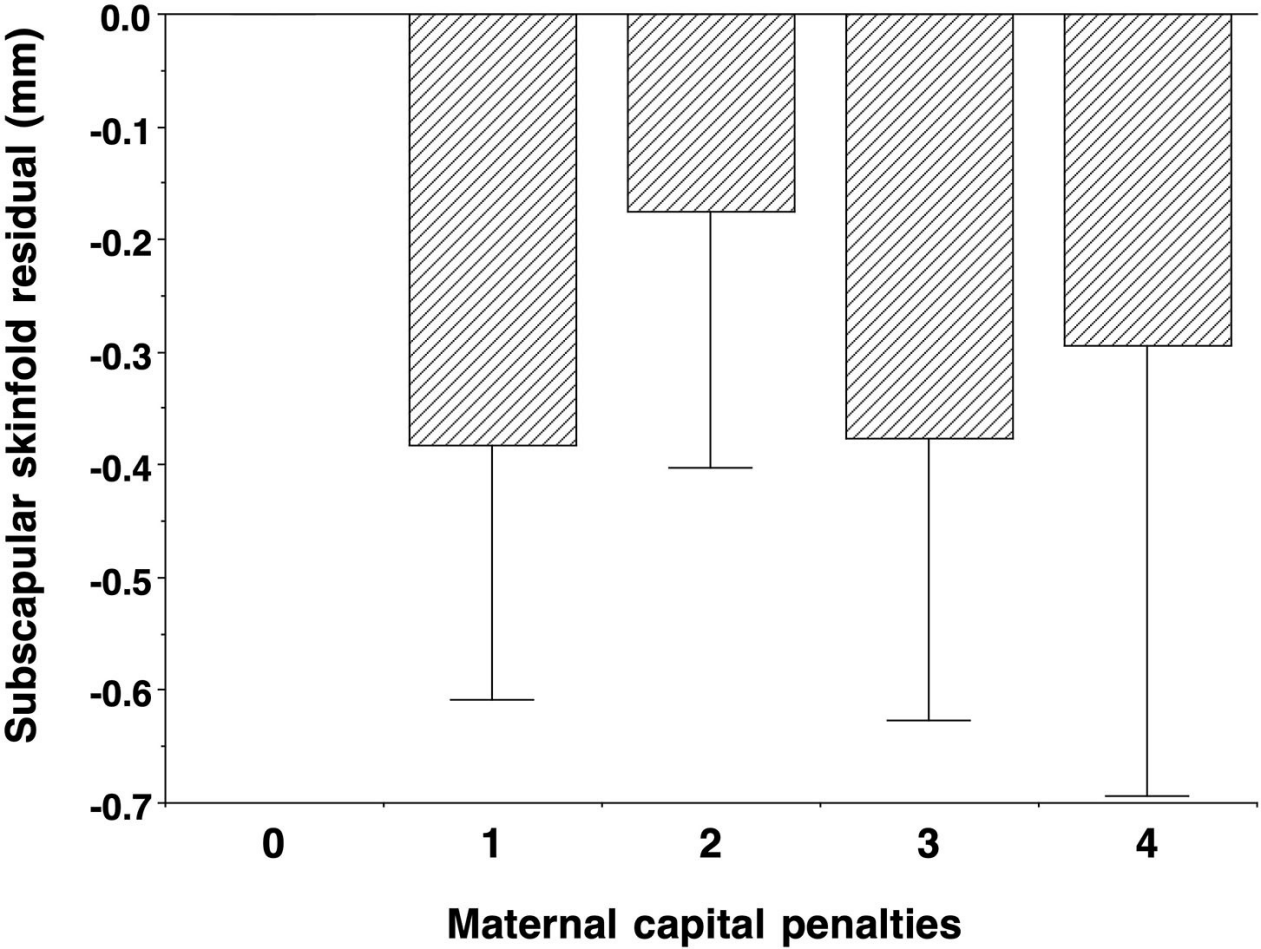
Dose-response associations of subscapular skinfold, adjusted for triceps skinfold, according to the number of “penalties” in maternal capital, relative to 0 penalties as the reference group. Lower maternal capital was not associated with a more central fat distribution. Test for trend *p* = 0.3.

Contrasting with the pattern seen for other outcomes, cardio-metabolic risk markers showed an unfavourable association with maternal capital: the higher the maternal capital, the higher the son's total cholesterol, HDL, LDL, triglycerides, and systolic BP, with a weaker trend also evident for diastolic BP. However, the associations for glucose, HbA1c, and the HDL/cholesterol ratio were null. When these associations were adjusted for the son's total fat mass, a clear trend remained evident only for HDL (Supplementary Table 4), indicating that adiposity was a strong mediator of the association of higher maternal capital with elevated cardio-metabolic risk. Adjusting for triceps skinfold, however, subscapular skinfold showed no association with maternal capital (*p* = 0.4), indicating that low maternal investment was not associated with a more central fat distribution (Figure 6).

### Clustering of adverse outcomes

We considered two possible drivers of clustering of adverse outcomes among the sons: early reproduction and low maternal capital. Regarding early reproduction, sons who had reproduced by 18 years comprised 7.4% of the population and accounted for 6.3% of obesity, 9.9% of short stature, 13% of violent crime, 18% of current smoking, and 19% of school dropout. Early reproduction, therefore, contributed only modestly to the clustering of adverse adult traits among sons, and the affected outcomes (ie those with greater frequency than expected given the numbers who had reproduced early) were all behavioural (violent crime, smoking, and school dropout). The one biological outcome that appeared in the cluster among early-reproducing sons was the low birth weight (12%), indicating that poor foetal growth might be a marker of early reproduction in later life.

To evaluate the second potential driver of clustering, maternal capital, we compared the sons with low maternal capital (3 or 4 penalties, *n* = 442), with the remainder of the cohort. The low capital group comprised 23% of the population and accounted for 14% of obesity, 24% of violent crime, 29% of smoking, 32% of school dropout, 35% of short stature, 39% of low birth weight, and 43% of early reproduction. Thus, compared to early reproduction, there was a greater degree of clustering of adverse outcomes in association with exposure to low maternal capital, and the outcomes affected spanned both biology (short stature, low birth weight) and behaviour (school dropout, smoking, violent crime, and early reproduction).

We then tested for independent associations of low maternal capital and early son reproduction with these adverse outcome variables, using logistic regression analysis. As shown in Table 4, both low maternal capital and early son reproduction independently predicted sons being out of school and being current smokers. Low maternal capital was associated with an increased risk of the son being of low birth weight and short stature, but a reduced risk of being overweight or obese, however early reproduction showed no association with short stature. Sons who had reproduced early were more likely to be receiving support, whereas low maternal capital was not associated with this outcome. Neither low maternal capital nor early reproduction was an independent predictor of the son having committed a violent crime.

**Table 4:** Multiple logistic regression models of independent associations of low maternal capital and early son reproduction with adverse son outcomes.

	**Low maternal capital**			**Early son reproduction**			
**Adverse outcome**	**OR**	**95% CI**	***p*-value**	**OR**	**95% CI**	***p*-value**	**Nagelkerke r^2^**
Studying last year	0.22	0.13, 0.35	<0.001	0.26	0.15, 0.45	<0.001	0.17
Studying now	0.27	0.20, 0.37	<0.001	0.28	0.15, 0.52	<0.001	0.17
Receiving support	0.75	0.55, 1.07	0.07	2.88	1.50, 5.51	<0.001	0.02
Paid for work	1.51	0.86, 2.66	0.1	1.99	0.60, 6.63	0.2	0.02
Smoked last week	1.77	1.20, 2.60	0.004	3.15	1.87, 5.28	<0.001	0.06
Current smoker	3.03	1.82, 5.04	<0.001	2.36	1.33, 4.19	0.003	0.08
Low birth weight	6.16	2.75, 13.79	<0.001	1.41	0.67, 2.95	0.3	0.09
Overweight	0.28	0.20, 0.40	<0.001	1.18	0.63, 2.21	0.5	0.10
Obesity	0.29	0.17, 0.49	<0.001	0.65	0.19, 2.17	0.4	0.07
Short stature	5.32	3.73, 7.60	<0.001	1.22	0.73, 2.05	0.4	0.18
Violent crime	1.05	0.72, 1.55	0.8	1.43	0.78, 2.63	0.2	0.00

Grey shading indicates association significant at p < 0.05.

Overall, these analyses support our earlier findings, that sons demonstrate primarily behavioural trade-offs in association with early reproduction, whereas exposure to low maternal capital is associated with both behavioural and physical trade-offs.

### Adjusting for direct mother-son transmission of traits

Given that early-reproducing sons were both shorter themselves and had shorter mothers, maternal phenotype should be controlled for when testing the association of early reproduction with the son's growth trajectory. Such associations might be driven by shared genetic factors or non-genetic inter-generational transmission. A similar scenario applies to behavioural traits, which might be replicated across generations due to shared family habits or opportunities.

Size at birth was not a predictor of early reproduction in bivariate analysis. However, after taking maternal weight into account, higher birth weight and BMI reduced the risk of the son reproducing early. Conversely, it was greater maternal stature, rather than the son's birth length, that showed a significant protective effect against the son's early reproduction (Table 5).

**Table 5:** Logistic regression models analysing the association of son's growth phenotype with the odds of early reproduction, without/with adjustment for maternal phenotype.

**Predictor**	**Model 1 (unadjusted)**		**Model 2 (adjusted)**	
	**Odds ratio**	**95% CI**	**Odds ratio**	**95% CI**
Birth weight (kg)	0.81	0.60, 1.11	0.62	0.48, 0.79
Maternal weight (kg)			0.98	0.97, 1.00
Weight 1 y (kg)	0.87	0.68, 1.11	0.90	0.69, 1.18
Maternal weight (kg)			1.01	0.94, 1.01
Weight 18 y (kg)	0.98	0.97, 0.99	0.99	0.97, 1.00
Maternal weight (kg)			0.97	0.97, 1.00
Birth length (cm)	0.98	0.92, 1.05	0.99	0.92, 1.06
Maternal height (cm)			0.97	0.95, 1.00
Length 1 y (cm)	0.95	0.86, 1.05	1.00	0.89, 1.11
Maternal height (cm)			0.92	0.88, 0.98
Height 18 y (cm)	0.95	0.93, 0.97	0.95	0.92, 0.98
Maternal height (cm)			0.99	0.97, 1.02
Birth BMI (kg/m^2^)	0.90	0.80, 0.94	0.90	0.78, 1.01
Maternal BMI (kg/m^2^)			0.98	0.93, 1.03
BMI 18 y (kg/m^2^)	0.98	0.80, 1.00	0.99	0.95, 1.03
Maternal BMI (kg/m^2^)			0.97	0.92, 1.02
Fat mass index 18 y (kg/m^2^)	0.93	0.88, 0.99	0.94	0.88, 1.00
Maternal BMI (kg/m^2^)			0.97	0.93, 1.02
Fat-free mas index 18 y (kg/m^2^)	1.08	0.99, 1.18	1.11	1.01, 1.22
Maternal BMI (kg/m^2^)			0.95	0.91, 1.00

BMI, body mass index.

n = 2,014 for all except weight at 1 year (n = 554) and length at 1 year (n = 546).

Grey shading indicates association significant at p < 0.05.

By adulthood, greater weight and height of the son were associated with a reduced risk of early reproduction, and this remained after adjustment for maternal size. Greater adiposity and fat-free mass also reduced the risk of early reproduction. These results persisted if adjusted for maternal capital: neither the son's adult weight nor fat mass predicted early reproduction, whereas both short stature and higher fat-free mass of the son were associated with increased odds (Supplementary Table 5). Overall, these findings suggest that maternal short stature indexed one pathway to the son's early reproduction, indicating an intergenerational effect, whereas the son's poor growth in childhood (but not in foetal life), indexed another pathway that was independent of maternal phenotype.

Similarly, we analysed whether early reproduction by the son predicted poor schooling outcomes, or the risk of being a smoker, taking into account maternal education and smoking in pregnancy respectively (Table 6). While lower maternal educational attainment was associated with the same outcome in the son, early reproduction by the son was also an independent predictor. Likewise, while maternal smoking was associated with son smoking, early reproduction by the son was also an independent predictor. These results persisted if adjusted for maternal capital score (Supplementary Table 6).

**Table 6:** Linear regression models analysing associations of early reproduction with components of son phenotype, without/with adjustment for the same or similar component of maternal phenotype.

**Outcome in son**	**Predictor**	**Model 1 (unadjusted)**		**Model 2 (adjusted)**	
		**Coeff**	**95% CI**	**Coeff**	**95% CI**
Years of education	Early reproduction	−1.64	−2.03, −1.25	−1.05	−2.40, −0.71
	Maternal education (y)			0.33	0.30, 0.35
		**OR**	**95% CI**	**OR**	**95% CI**
Studying now	Early reproduction	0.17	0.11, 0.26	0.20	0.13, 0.32
	Maternal education 1			0.28	0.22, 036
	Maternal education 2			0.42	0.34, 0.52
Current smoker	Early reproduction	3.56	2.49, 5.10	3.36	2.31, 4.84
	Maternal smoking			1.84	1.43, 2.37

Maternal education group 1: 0–4 years, group 2: 5–7 years; reference group 8+ years.

Maternal income group 1: 0–2 minimum wages, group 2: 3–4 minimum wages, reference group 5+ minimum wages.

n = 2,014.

Grey shading indicates association significant at p < 0.05.

In general, therefore, early reproduction was associated with unfavourable trade-offs relating to childhood growth, health, education, and risk-taking, independent of the potential direct maternal transmission of phenotypic traits.

## Discussion

**Figure 7:**
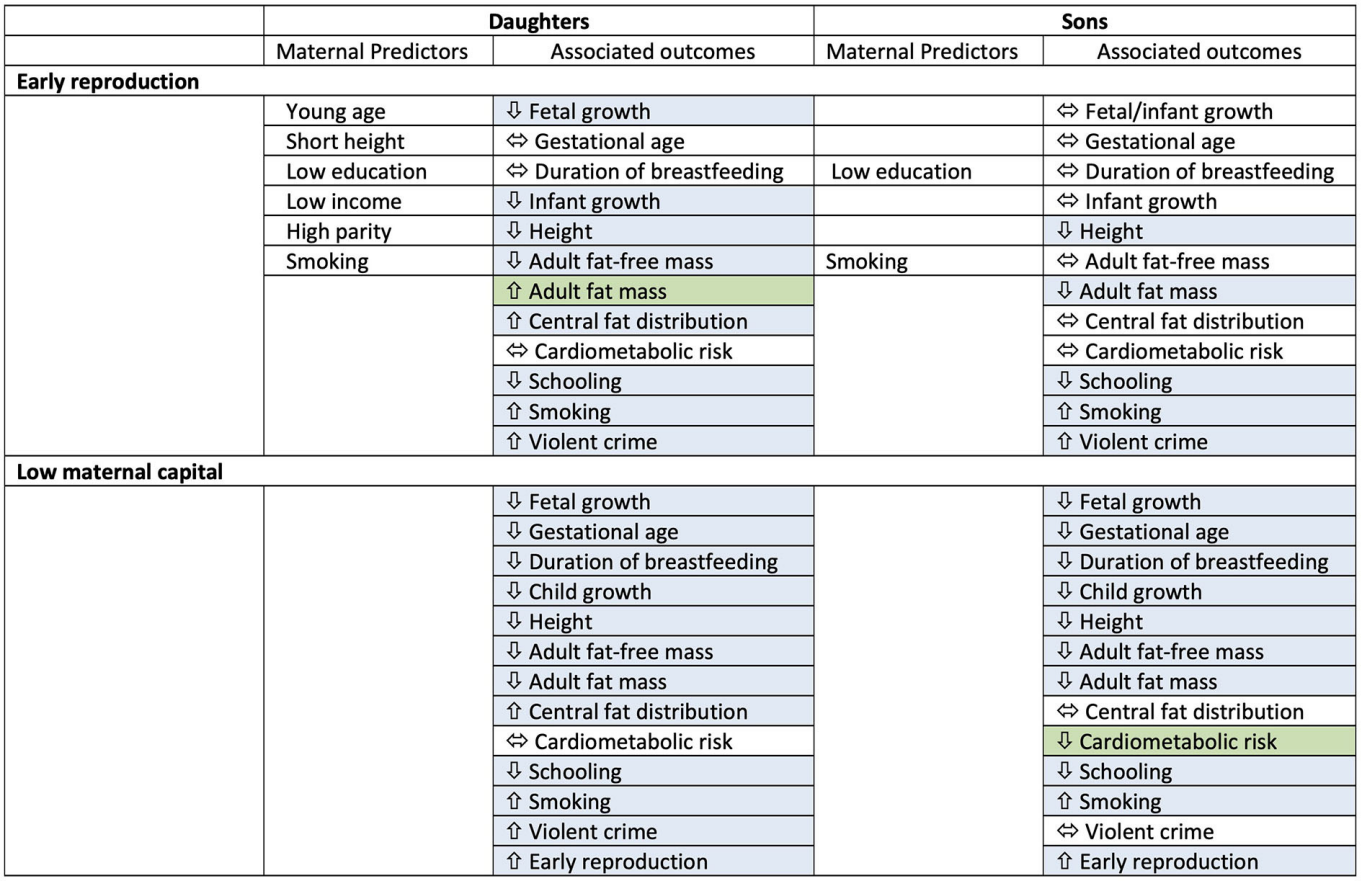
Summary table for results of associations of early reproduction and exposure to low maternal capital with outcomes in both sons and daughters. Maternal predictors of early reproduction are also indicated. Blue cells indicate an association, green cells indicate where one sex shows the opposite direction of effect compared to the other sex. Overall, sons show fewer associations of physical traits with early reproduction, and in contrast to daughters, low maternal capital is associated with lower cardiometabolic risk in sons.

As in daughters (14), our analysis of sons has shown that exposure to low maternal capital in early life is associated with future discounting, indicated by trade-offs between competing life history functions of growth, metabolic health, risky behaviour, and education and work. More explicitly, early life adversity is associated with prioritising early reproduction at the expense of investing in growth or other aspects of human capital. These trade-offs associated with maternal capital levels might emerge, depending on the outcome, either through behavioural decisions of the offspring, through physiological responses involving no conscious deliberation, or through a combination of both mechanisms. However, at a more detailed level, the pattern of trade-offs showed several differences compared to those of daughters, indicating that sons and daughters respond differently to low levels of maternal investment (Figure 7). Moreover, the role of early reproduction in mediating the association of low maternal capital with adverse outcomes in the offspring is weaker in sons compared to daughters, and sons pay fewer physical costs in association with early reproduction.

A key finding, similar to that in daughters (14), was that exposure to low maternal capital was associated with an increased likelihood of early reproduction by sons. Only 3% of sons in the highest maternal capital group reproduced early, compared to 17% in the lowest group. As this is a key tenet of life history theory—that less investment in early life, a marker of reduced longevity, favours allocating resources away from other life history functions towards earlier reproduction (27)—we first evaluated how reproduction itself was associated with adverse outcomes. Similar to our findings for daughters, early reproduction by sons were associated with poorer physical growth, less education, and more risky behaviour. This indicates that those who grew poorly in early life were more likely to reproduce early. However, unlike in the daughters, the reduced investment in physical growth did not occur in foetal life or infancy, and instead emerged only during childhood. Moreover, unlike in daughters, early reproduction was not associated with a more central fat distribution, a marker of investment in immune defence. Overall, early reproduction in sons appeared to involve little physical cost, whereas daughters who reproduced early demonstrated poor quality growth much earlier in the life course (foetal life) and more extensive somatic penalties in adult life (14).

Another contrast between the sexes was that almost every individual marker of low maternal capital was independently associated with an increased likelihood of daughters reproducing early, whereas only low maternal education and maternal smoking in pregnancy were independent predictors of sons reproducing early. Maternal parity 3+ was not a predictor of early reproduction, whereas parity 5+ was, indicating that earlier reproducing sons tended to come from relatively large families, and might themselves have wanted to have started a family earlier, a finding reported in a UK study of adolescent women (41). Overall, it appears that the “pathway” to early reproduction is less sensitive to maternal physical traits in sons than daughters and that the key period of exposure for sons may be childhood rather than foetal life.

In daughters in this cohort (14), similar to other studies on girls (42, 43), school dropout and early pregnancy were related. In girls, these outcomes demonstrate a two-way street, whereby poor school performance may lead to early reproduction, while early pregnancy may cause girls to leave school (44, 45). Fewer studies have addressed this dynamic in boys, but some studies find similar associations of poor school performance with boys' early marriage or the initiation of sexual behaviour (46–48). In this cohort, we replicated our findings for daughters, showing that early reproduction in sons was associated with less education, and an increased likelihood of two types of risky behaviour (smoking, violent crime). Such correlations of adverse behavioural outcomes have been reported in other studies and settings (15, 49, 50), and have also been explored using an evolutionary life history lens in other species (51). As with the daughters (14), however, early-reproducing sons were more likely to consider education important compared to their non-reproducing peers and were more likely to cite work as the main reason for being out of school and to be earning wages from paid labour. Whereas the daughters in this cohort traded off school directly with bearing children (14), the sons, therefore, demonstrated a trade-off between learning (with potentially greater future returns) and more immediate wage-earning. This is consistent with men's lack of direct parental investment in pregnancy and lactation, indicating that they require other strategies for parental investment (52).

Overall, therefore, early reproduction contributed primarily to the clustering of a set of adverse behavioural outcomes among sons (school dropout, smoking, and violent crime) but was poorly predictive of adverse physiological outcomes. This may indicate that early-reproducing sons live in households that already demonstrate “future discounting” at a behavioural level, and replicate this pattern in their own life-course. Low maternal education was the strongest predictor of the sons reproducing early and was also an independent predictor in daughters. This may indicate an intergenerational pattern of reduced schooling, whereby women trade off education for early reproduction, and similar trade-offs are then made by their offspring of both sexes. Nevertheless, we emphasise that the majority of those not in school did not indicate that they considered education unimportant; the main reason given for having left school being either having children (women: 69%; men: 10%) or working (men: 62%).

These findings help explain the associations of maternal capital with sons' life history trade-offs. Low maternal capital was associated with sons' poor growth from foetal life onwards, and with smaller body size, and lower fat and fat-free mass in adulthood. Individual associations of maternal traits (eg nutritional status, education, wealth, smoking) with physical growth patterns in both sexes have been widely reported previously (35, 53–56). Several studies from low and middle-income countries have shown that higher birth weight is associated with greater FFM in males, but with greater fat mass in females (57), however, we observed similar associations of low maternal capital with reduced FFM and fat mass patterns in both sexes in this cohort. Exposure to lower maternal capital was also associated with a higher risk of school dropout, early reproduction, and smoking, but not with financial income or violent crime. Other studies have linked maternal undernutrition, smoking, and lack of education with adverse social outcomes in the offspring, involving both physical and behavioural mechanisms (45, 58–61). Overall in our study, exposure to low maternal capital in early life was more successful in explaining both physical (low birth weight, short stature) and behavioural traits, compared to early reproduction which explained only behavioural outcomes.

For daughters, we previously found that neither early reproduction nor exposure to low maternal capital was strongly predictive of cardio-metabolic risk profile, except for central fatness which was elevated in both risk groups (14). Although several markers of higher metabolic risk (low birth weight, short stature, obesity, and central fat) were evident for daughters reproducing early or exposed to low maternal capital, this did not propagate to the direct metabolic measurements, and in some cases, risk markers such as cholesterol and triglycerides were lower in the groups that had invested more in survival/reproduction. In sons, the association of early reproduction with cardio-metabolic risk was again broadly null, though markers of adiposity were lower in early-reproducing sons. However, there was a much stronger pattern for maternal capital, whereby the lower the level of maternal capital the lower the son's cardio-metabolic risk profile. The only traits that did not show this finding were markers of glycemic control (glucose, HbA1c) and central fat distribution.

This indicates that while the sons of high capital mothers may achieve better growth and adult size, and delay the onset of reproduction to capitalise on these traits (potentially benefitting from the opportunity to reproduce with partners of higher socio-economic status), they do so at a cost of poorer cardio-metabolic health. This may be in part explained by the greater opportunity for wealthier families to access palatable but fattening processed foods, and hence higher body fat levels, as widely reported in Brazil (62). These effects are strongly mediated by higher overall levels of fat, but not a more central fat distribution. From a life history perspective, high capital sons have greater energy reserves but have not allocated them disproportionately to immune defence.

We emphasise that at this age, overt levels of cardio-metabolic risk are low in this population; for example, only 2% of the sons had hypertension as defined by diastolic blood pressure. In the longer term, sons and daughters of low capital mothers have lower birth weight and shorter stature, while also being more likely to smoke. According to the logic of the “capacity-load” model (63), this may make them more susceptible to non-communicable diseases in later life, especially if they become overweight. Our findings, therefore, suggest that the pathway to elevated cardio-metabolic risk may be different, depending on exposure to maternal capital and developmental trajectory. High capital sons have elevated fatness (metabolic load) but also developed better metabolic capacity in early life. Low capital sons have a low metabolic load but developed poorer metabolic capacity in early life. In turn, different interventions may be required to reduce long-term disease risk.

Our results highlight an important sex difference in the way adiposity mediates physical trade-offs. Generically, females have more body fat than males, particularly at around 18 years. In both sexes, exposure to low maternal capital was associated with less total body fat, but whereas this was associated with a more central fat distribution in daughters, it was not in sons. The contrast was even greater for early reproduction: early reproducing daughters had more body fat than their non-reproducing peers who had not reproduced, though lower triceps skinfold, and again a more central fat distribution. For women, body fat is well-established to fund lactation, and hence benefits reproductive investment (24). Conversely, early reproducing sons had substantially less body fat than their non-reproducing peers, and no evidence of a more central fat distribution, and they had preserved their fat-free mass, which has been associated with reproductive success in men (26). From a life history perspective, this indicates reduced resilience against future infections/famines in low capital and early reproducing sons, and hence future discounting similar to that demonstrated by the clustering of early reproduction, school dropout, and smoking.

Our markers of risky behaviour (smoking, violent crime) are best considered not as direct investment in any of the four life history functions, but rather as an indication of future discounting (13). Smoking reduces maintenance in health and may potentially reduce longevity, but in the short term may be used as a palliative to mitigate psychosocial stress. Violent crime might produce short-term pay-offs at cost of the longer-term capacity to invest in offspring (the possibility of social punishment such as prison). Of particular relevance, levels of the hormone testosterone have been linked with aggressive behaviour and violent crime in men (64, 65), and the same hormone has well-established associations with secondary sexual characteristics and sexual behaviour (66, 67).

Our analyses of early reproduction included adjustment for maternal traits, to improve confidence that sons' trade-offs were a direct consequence of their own developmental trajectory and not simply the replication of maternal phenotype. The findings broadly supported this hypothesis, but this does not mean that genetic susceptibility to adverse outcomes plays no role. Recent analysis suggests that key early life “environmental” influences, relating to parents or neighbourhoods, also correlate with genetic markers of the child's propensity to adverse outcomes, examples including alleles associated with education, BMI, and schizophrenia (68). In our analysis, an intriguing finding was that maternal short stature indexed one pathway to the son's early reproduction, indicating an intergenerational effect, whereas the son's poor growth in childhood indexed an additional pathway that was independent of maternal phenotype. Further work will be needed to explore the contribution of genetic and environmental factors to the kinds of trade-offs we have reported.

## Strengths and limitations

Many of the strengths and limitations of this analysis are the same as those we described in detail for the daughters' analysis (14). Briefly, strengths include the rich dataset and the large sample size from a middle-income country, and the prospective collection of data on maternal capital and the offspring in early life. Moreover, a major strength is the opportunity to interrogate maternal investment and developmental and reproductive outcomes in men, as the majority of work to date has focused on women.

Among the limitations are the 22% cohort attrition by 18 years, small amounts of missing data, the lack of data on paternal phenotype or the overall reproductive career of the mothers, and the fact that our analysis is necessarily observational, meaning that we cannot demonstrate causality in the trade-offs we describe. As with the daughters' analyses, none of these limitations is substantial, indicating that our findings are likely to be robust.

A different limitation, of particular relevance to the current analysis, is that since men start to reproduce later than women on average, a direct comparison of men and women at 18 years is difficult to interpret. Whereas 15% of the cohort of women had a child by 18 years of age (14), only 7% of the men did. However, since the cohort was followed up at 18 years, this was the only comparison that was possible. Moreover, the sample size of 150 men was still adequate to identify some predictors and some associated outcomes. A contrasting limitation is that not all men may have been willing to admit in the questionnaire that they had been responsible for a pregnancy. This may make our findings conservative.

## Conclusions

In conclusion, we found that, as in the daughters in this birth cohort, both early reproduction and exposure to markers of low maternal investment are associated with phenotypic patterns in sons that indicate trade-offs between life history functions. Our work, therefore, helps understand why adverse outcomes may cluster within susceptible subsets in a population, with low maternal capital appearing as a more important driver of this clustering than early reproduction. Our primary finding was that, as in daughters, the patterns indicated a degree of future discounting, with both reduced maternal investment and early reproduction associated with less investment in growth and maintenance along with greater risky behaviour. Lower maternal investment was associated with a greater likelihood of early reproduction by sons.

However, we also found several notable contrasts compared to our earlier analyses of daughters. First, early reproduction appeared to be a weaker mediator of the associations of low maternal capital with adverse sons outcomes. Second, sons paid fewer physical costs in association with early reproduction. Third, sons with high-capital mothers had worse cardio-metabolic outcomes, mediated by higher fatness; however, in the longer-term, sons of low-capital mothers may still be more susceptible to both infectious and non-communicable diseases.

Overall, sons appear to suffer fewer penalties than daughters in association with reduced maternal investment. Sons of low capital mothers were shorter and leaner than their high capital peers but had managed to preserve their lean mass, a trait of value in male reproduction. They had avoided cardio-metabolic costs, but also had lower energy reserves, potentially constraining immune function. They were less educated but had transitioned to wage work which could offer immediate opportunities to invest in their families. Further work is needed to investigate how these trade-offs accumulate through the life-course, as cohort members complete their reproduction and become at greater risk of overt non-communicable disease in association with ageing.

Our results have several implications for policy. First, low maternal capital accounted for a substantial degree of clustering of adverse outcomes among sons. Interventions to promote maternal capital may therefore represent the most effective pathway to improve sons' outcomes, as indicated also for daughters (14). Maternal capital is the first environmental niche to which the offspring is exposed, and responses to all other environmental exposures are made under the imprint of this initial developmental exposure (69).

Second, to improve offspring outcomes comprehensively, both multi-sectoral and specific education and health interventions are needed, ideally initiated before women conceive and persisting during the early critical windows of offspring development (70–74). This composite and early approach may be more effective in disrupting the accumulation of penalties across the life-course than interventions that are provided only during childhood and adolescence (15).

Third, to be most effective in breaking inter-generational cycles of disadvantage, interventions should target both mothers and their offspring, as post-natal interventions (promoting exclusive breast-feeding, immunizations, education etc) may be able to ameliorate some of the adverse consequences of exposure to poor maternal nutritional status *in utero*. In that context, broader societal gender inequality must also be addressed, because of its cumulative adverse impacts on the offspring through the life-course. Importantly, gender inequality has been linked with adverse outcomes in offspring of both sexes, including low birth weight, early child development, child malnutrition and mortality risk, and attitudes to education (75–78).

However, given that the associations of maternal factors with outcomes varied between daughters and sons (Figure 7), we should recognise that the benefits of interventions to improve maternal capital may also differ by offspring sex, because of inherent biological differences between boys and girls that have been shaped by natural or sexual selection, and which drive differential investment across life history functions. This hypothesis requires testing using an experimental approach such as individual level or cluster randomised control trials. From an intergenerational perspective, moreover, many interventions have targeted adolescent girls as “future mothers” to improve components of maternal capital (79, 80), but much less is known about the potential benefits to offspring health of targeting boys as “future fathers”, to improve paternal capital.

## Data availability statement

The raw data supporting the conclusions of this article will be made available by the authors for the purposes of replicating these analyses, without undue reservation.

## Ethics statement

The studies involving human participants were reviewed and approved by Medical School Ethics Committee of the Federal University of Pelotas. Written informed consent to participate in this study was provided by the participants' legal guardian/next of kin.

## Author contributions

JW, TC, MC-B, and AAM designed the analysis. The Pelotas 1993 birth cohort is overseen by AMM, who steered the 18-year follow-up conducted by FW, PO, HG, and IO. Initial statistical analysis was undertaken by JW, TC, and MC-B and revised in light of feedback from AMM, JM, AAM, RS, and DL. All authors reviewed draughts, provided critical feedback, and approved the final manuscript.

## Funding

This work was supported by the Science and Technology Department, Brazilian Ministry of Health, with resources transferred through the Brazilian National Council for Scientific and Technological Development (CNPq) [Grant Number 400943/2013-1]. The study Pelotas Birth Cohort, 1993 was conducted by the Postgraduate Program in Epidemiology at Universidade Federal de Pelotas, with the collaboration of the Brazilian Public Health Association (ABRASCO). From 2004 to 2013, the Wellcome Trust supported the 1993 Birth Cohort study [Grant Number 086974/Z/08/Z]. The initial phases of the cohort were funded by the European Union and the Brazilian National Program for Centres of Excellence (PRONEX), the National Research Council (CNPq), and the Ministry of Health. This research benefitted from funding awarded to the NIHR Great Ormond Street Hospital Biomedical Research Centre. The study sponsors played no part in the design, data analysis, and interpretation of this study; the writing of the article or the decision to submit the paper for publication, and the authors' work was independent of their funders.

## Conflict of interest

The authors declare that the research was conducted in the absence of any commercial or financial relationships that could be construed as a potential conflict of interest.

## Publisher's note

All claims expressed in this article are solely those of the authors and do not necessarily represent those of their affiliated organizations, or those of the publisher, the editors and the reviewers. Any product that may be evaluated in this article, or claim that may be made by its manufacturer, is not guaranteed or endorsed by the publisher.

## References

[1] Stearns SC The The Evolution of Life Histories. Oxford: Oxford University Press (1992).

[2] Hill K. Life history theory and evolutionary anthropology. Evol Anthropol. (1993) 2:78–89. 10.1002/evan.1360020303

[3] Barker DJ. The developmental origins of chronic adult disease. Acta Paediatr Suppl. (2004) 93:26–33. 10.1111/j.1651-2227.2004.tb00236.x 15702667

[4] Wells JC Sawaya AL Wibaek R Mwangome M Poullas MS Yajnik CS. The double burden of malnutrition: etiological pathways and consequences for health. Lancet. (2019) 395:75–88. 10.1016/S0140-6736(19)32472-9 31852605 PMC7613491

[5] Entringer S Buss C Wadhwa PD. Prenatal stress and developmental programming of human health and disease risk: concepts and integration of empirical findings. Curr Opin Endocrinol Diabetes Obes. (2010) 17:507–16. 10.1097/MED.0b013e3283405921 20962631 PMC3124255

[6] Barker DJ Osmond C Forsen TJ Kajantie E Eriksson JG. Trajectories of growth among children who have coronary events as adults. N Engl J Med. (2005) 353:1802–9. 10.1056/NEJMoa044160 16251536

[7] Bhargava SK Sachdev HS Fall CH Osmond C Lakshmy R Barker DJ. Relation of serial changes in childhood body-mass index to impaired glucose tolerance in young adulthood. N Engl J Med. (2004) 350:865–75. 10.1056/NEJMoa035698 14985484 PMC3408694

[8] Marmot M. Social determinants of health inequalities. Lancet. (2005) 365:1099–104. 10.1016/S0140-6736(05)71146-6 15781105

[9] Kinner SA Borschmann R. Inequality and intergenerational transmission of complex adversity. Lancet Public Health. (2017) 2:PE342–343. 10.1016/S2468-2667(17)30139-1 29253468

[10] Boullier M Blair M. Adverse chilhood experiences. Paediatr Child Health. (2018) 28:132–7. 10.1016/j.paed.2017.12.008

[11] Gluckman PD Hanson MA Cooper C Thornburg KL. Effect of in utero and early-life conditions on adult health and disease. N Engl J Med. (2008) 359:61–73. 10.1056/NEJMra0708473 18596274 PMC3923653

[12] Wells JCK Nesse RM Sear R Johnstone RA Stearns SC. Evolutionary public health: introducing the concept. Lancet. (2017) 390:500–9. 10.1016/S0140-6736(17)30572-X 28792412

[13] Schechter DE Francis CE. A life history approach to understanding youth time preference. Hum Nat. (2010) 21:140–64. 10.1007/s12110-010-9084-2

[14] Wells JCK Cole TJ Cortina-Borja M Sear R Leon DA Marphatia AA. Low maternal capital predicts life history trade-offs in daughters: why adverse outcomes cluster in individuals. Front Public Health. (2019) 7:206. 10.3389/fpubh.2019.00206 31417889 PMC6685417

[15] Caspi A Houts RM Belsky DW Harrington H Hogan S Ramrakha S. Childhood forecasting of a small segment of the population with large economic burden. Nat Hum Behav. (2016) 1:0005. 10.1038/s41562-016-0005 28706997 PMC5505663

[16] Richmond-Rakerd LS D'Souza S Andersen SH Hogan S Houts RM Poulton R. Clustering of health, crime and social-welfare inequality in 4 million citizens from two nations. Nat Hum Behav. (2020) 4:255–64. 10.1038/s41562-019-0810-4 31959926 PMC7082196

[17] Kirkwood TBL Rose MR. Evolution of senescence. Phil Tran R Soc Lond B. (1991) 332:15–24. 10.1098/rstb.1991.0028 1677205

[18] McDade TW Chyu L Duncan GJ Hoyt LT Doane LD Adam EK. Adolescents' expectations for the future predict health behaviors in early adulthood. Soc Sci Med. (2011) 73:391–8. 10.1016/j.socscimed.2011.06.005 21764487 PMC3148854

[19] Wells JC. Maternal capital and the metabolic ghetto: An evolutionary perspective on the transgenerational basis of health inequalities. Am J Hum Biol. (2010) 22:1–17. 10.1002/ajhb.20994 19844897

[20] Wells JC. The Metabolic Ghetto: An Evolutionary Perspective on Nutrition, Power Relations and Chronic Disease. Cambridge: Cambridge University Press (2016).

[21] Kaplan H Lancaster J Robson A. Embodied capital and the evolutionary economics of the human life span. In: Carey JR Tuljapurkar S, editors. Life Span: Evolutionary, Ecological. New York, NY: Demographic Perspectives Population Council (2003).

[22] Sear R Sheppard P Coall DA. Cross-cultural evidence does not support universal acceleration of puberty in father-absent households. Philos Trans R Soc Lond B Biol Sci. (2019) 374:20180124. 10.1098/rstb.2018.0124 30966893 PMC6460089

[23] Dufour DL Sauther ML. Comparative and evolutionary dimensions of the energetics of human pregnancy and lactation. Am J Hum Biol. (2002) 14:584–602. 10.1002/ajhb.10071 12203813

[24] Wells JCK. Life history trade-offs and the partitioning of maternal investment: Implications for health of mothers and offspring. Evol Med Public Health. (2018) 2018:153–66. 10.1093/emph/eoy014 30152817 PMC6101534

[25] Wells JC. Sexual dimorphism of body composition. Best Pract Res Clin Endocrinol Metab. (2007) 21:415–30. 10.1016/j.beem.2007.04.007 17875489

[26] Lassek WD Gaulin SJ. Costs and benefits of fat-free muscle mass in men: relationship to mating success, dietary requirements, native immunity. Evol Hum Behav. (2009) 5:322–8. 10.1016/j.evolhumbehav.2009.04.002

[27] Walker R Gurven M Hill K Migliano A Chagnon N De SR. Growth rates and life histories in twenty-two small-scale societies. Am J Hum Biol. (2006) 18:295–311. 10.1002/ajhb.20510 16634027

[28] Sheppard P Sear R. Father absence predicts age at sexual maturity and reproductive timing in British men. Biol Lett. (2012) 8:237–40. 10.1098/rsbl.2011.0747 21900315 PMC3297378

[29] Sheppard P Garcia JR Sear R. A not-so-grim tale: how childhood family structure influences reproductive and risk-taking outcomes in a historical US Population. PLoS One. (2014) 9:e89539. 10.1371/journal.pone.0089539 24599234 PMC3943735

[30] Sheppard P Garcia JR Sear R. Childhood family disruption and adult height: is there a mediating role of puberty? Evol Med Public Health. (2015) 2015:332–42. 10.1093/emph/eov028 26609061 PMC4697772

[31] Goncalves H Assuncao MC Wehrmeister FC Oliveira IO Barros FC Victora CG. Cohort profile update: the 1993 Pelotas (Brazil) birth cohort follow-up visits in adolescence. Int J Epidemiol. (2014) 43:1082–8. 10.1093/ije/dyu077 24729426 PMC4121560

[32] Victora CG Hallal PC Araujo CL Menezes AM Wells JC Barros FC. Cohort profile: the 1993 Pelotas (Brazil) birth cohort study. IntJ Epidemiol. (2008) 37:704–9. 10.1093/ije/dym177 17846051

[33] Goncalves H Wehrmeister FC Assuncao MCF Tovo-Rodrigues L Oliveira IO Murray J. Cohort profile update: the 1993 Pelotas (Brazil) birth cohort follow-up at 22 years. Int J Epidemiol. (2018) 47:1389–1390e. 10.1093/ije/dyx249 29240909 PMC6208268

[34] United Nations Children's Fund. Ending Child Marriage: Progress and Prospects. New York, NY: UNICEF (2014).

[35] Ozaltin E Hill K Subramanian SV. Association of maternal stature with offspring mortality, underweight, and stunting in low- to middle-income countries. Jama. (2010) 303:1507–16. 10.1001/jama.2010.450 20407060 PMC3100588

[36] Victora CG Barros FC Vaughan JP Martines JC Beria JU. Birthweight, socio-economic status and growth of Brazilian infants. Ann Hum Biol. (1987) 14:49–57. 10.1080/03014468700008831 3592612

[37] Dharmalingam A Navaneetham K Krishnakumar CS. Nutritional status of mothers and low birth weight in India. Matern Child Health J. (2010) 14:290–8. 10.1007/s10995-009-0451-8 19199015

[38] Makoka D Masibo PK. Is there a threshold level of maternal education sufficient to reduce child undernutrition? Evidence from Malawi, Tanzania and Zimbabwe. BMC Pediatr. (2015) 15:96. 10.1186/s12887-015-0406-8 26297004 PMC4546212

[39] Hales CN Barker DJ. Type 2 (non-insulin-dependent) diabetes mellitus: the thrifty phenotype hypothesis. Diabetologia. (1992) 35:595–601. 10.1007/BF00400248 1644236

[40] Cole TJ Altman DG. Statistics Notes: Percentage differences, symmetry, natural logarithms. BMJ. (2017) 358:j3683. 10.1136/bmj.j3683 28814563

[41] Nettle D Dickins TE Coall DA de Mornay Davies P. Patterns of physical and psychological development in future teenage mothers. Evol Med Public Health. (2013) 2013:187–96. 10.1093/emph/eot016 24481198 PMC3868355

[42] Marphatia AA Saville NM Amable GS Manandhar DS Cortina-Borja M Wells JC. How much education is needed to delay women's age at marriage and first pregnancy? Front Public Health. (2019) 7:396. 10.3389/fpubh.2019.00396 31993411 PMC6964653

[43] UNESCO. Early and Unintended Pregnancy and the Education Sector: Evidence Review. Paris: UNESCO (2017).

[44] Birchall J. Early Marriage, Pregnancy and Girl Child School Dropout. K4D Helpdesk Report. Brighton: Institute of Development Studies (2018).

[45] Marphatia AA Wells JC Reid AM Yajnik CS. Biosocial life-course factors associated with women's early marriage in rural India: The prospective longitudinal Pune Maternal Nutrition Study. Am J Biol Anthropol. (2022) 177:147–61. 10.1002/ajpa.24408 36787733

[46] Dearden K Hale C Alvarez J. The educational antecedents of teen fatherhood. Br J Educ Psychol. (1992) 62:139–47. 10.1111/j.2044-8279.1992.tb01007.x 1558810

[47] Glynn JR Sunny BS DeStavola B Dube A Chihana M Price AJ. Early school failure predicts teenage pregnancy and marriage: a large population-based cohort study in northern Malawi. PLoS ONE. (2018) 13:e0196041. 10.1371/journal.pone.0196041 29758040 PMC5951561

[48] Sunny BS DeStavola B Dube A Price A Kaonga AM Kondowe S. Lusting, learning and lasting in school: sexual debut, school performance and dropout among adolescents in primary schools in Karonga district, northern Malawi. J Biosoc Sci. (2019) 51:720–36. 10.1017/S0021932019000051 31030681

[49] Caspi A Begg D Dickson N Harrington H Langley J Moffitt TE. Personality differences predict health-risk behaviors in young adulthood: evidence from a longitudinal study. J Pers Soc Psychol. (1997) 73:1052–63. 10.1037/0022-3514.73.5.1052 9364760

[50] Zuckerman M Kuhlman DM. Personality and Risk-Taking: Common Bisocial Factors. J Pers. (2000) 68:999–1029. 10.1111/1467-6494.00124 11130742

[51] Wolf Leimar O Weissing FJ. Life-history trade-offs favour the evolution of animal personalities. Nature. (2007) 447:581–4. 10.1038/nature05835 17538618

[52] Geary DC. Evolution of paternal investment. In: Buss DM, editor. The Evolutionary Psychology Handbook. Hoboken, NJ: Wiley & Sons (2005). p. 483–505.

[53] Jeong J Kim R Subramanian SV. How consistent are associations between maternal and paternal education and child growth and development outcomes across 39 low-income and middle-income countries? J Epidemiol Community Health. (2018) 72:434–41. 10.1136/jech-2017-210102 29439191

[54] Lee HY Song IH Kawachi I. Maternal and child social support and food availability in relation to child growth in four low- and middle-income countries. Sci Rep. (2022) 12:5910. 10.1038/s41598-022-09850-1 35396562 PMC8993861

[55] Prasad JB Pezhhan A Patil SH. Effect of wealth, social inequality, Mother's BMI, and education level on child malnutrition in India. Diabetes Metab Syndr. (2021) 15:102304. 10.1016/j.dsx.2021.102304 34601344

[56] Leary SD Smith GD Rogers IS Reilly JJ Wells JC Ness AR. Smoking during pregnancy and offspring fat and lean mass in childhood. Obesity. (2006) 14:2284–93. 10.1038/oby.2006.268 17189557 PMC1890311

[57] Wells JC Chomtho S Fewtrell MS. Programming of body composition by early growth and nutrition. ProcNutrSoc. (2007) 66:423–34. 10.1017/S0029665107005691 17637095

[58] Victora CG Adair L Fall C Hallal PC Martorell R Richter L. Maternal and child undernutrition: consequences for adult health and human capital. Lancet. (2008) 371:340–57. 10.1016/S0140-6736(07)61692-4 18206223 PMC2258311

[59] Kandel DB Griesler PC Schaffran C. Educational attainment and smoking among women: risk factors and consequences for offspring. Drug Alcohol Depend. (2009) 104 (Suppl. 1):S24–33. 10.1016/j.drugalcdep.2008.12.005 19179020 PMC2774716

[60] Ayano G Betts K Dachew BA Alati R. Maternal smoking during pregnancy and poor academic performance in adolescent offspring: a registry data-based cohort study. Addict Behav. (2021) 123:107072. 10.1016/j.addbeh.2021.107072 34364108

[61] Marphatia AA Reid AM Yajnik CS. Developmental origins of secondary school dropout in rural India and its differential consequences by sex: a biosocial life-course analysis. Int J Educ Dev. (2019) 66:8–13. 10.1016/j.ijedudev.2018.12.001

[62] Monteiro CA Levy RB Claro RM de Castro IR Cannon G. Increasing consumption of ultra-processed foods and likely impact on human health: evidence from Brazil. Public Health Nutr. (2011) 14:5–13. 10.1017/S1368980010003241 21211100

[63] Wells JCK. The capacity-load model of non-communicable disease risk: understanding the effects of child malnutrition, ethnicity and the social determinants of health. Eur J Clin Nutr. (2018) 72:688–97. 10.1038/s41430-018-0142-x 29748656

[64] Dabbs JM Carr TS Frady RL Riad JK. Testosterone, crime, and misbehavior among 692 male prison inmates. Pers Individ Dif. (1995) 18:627–83. 10.1016/0191-8869(94)00177-T

[65] Batrinos ML. Testosterone and aggressive behavior in man. Int J Endocrinol Metab. (2012) 10:563–8. 10.5812/ijem.3661 23843821 PMC3693622

[66] Peters M Simmons L Rhodes G. Testosterone is associated with mating success but not attractiveness or masculinity in human males. Anim Behav. (2008) 76:297–303. 10.1016/j.anbehav.2008.02.008

[67] Bogaert A Fisher W. Predictors of university men's number of sexual partners. J Sex Res. (1995) 32:119–30. 10.1080/00224499509551782

[68] Krapohl E Hannigan LJ Pingault JB Patel H Kadeva N Curtis C. Widespread covariation of early environmental exposures and trait-associated polygenic variation. Proc Natl Acad Sci U S A. (2017) 114:11727–32. 10.1073/pnas.1707178114 29078306 PMC5676894

[69] Wells JC. Obesity as malnutrition: the role of capitalism in the obesity global epidemic. Am J Hum Biol. (2012) 24:261–76. 10.1002/ajhb.22253 22383142

[70] Victora CG Hartwig FP Vidaletti LP Martorell R Osmond C Richter LM. Effects of early-life poverty on health and human capital in children and adolescents: analyses of national surveys and birth cohort studies in LMICs. Lancet. (2022) 399:1741–52. 10.1016/S0140-6736(21)02716-1 35489358 PMC9061872

[71] Maluccio JA Hoddinott J Behrman JR Martorell R Quisumbing AR Stein AD. The impact of improving nutrition during early childhood on education among Guatemalan adults. Economic Journal. (2009) 119:734–63. 10.1111/j.1468-0297.2009.02220.x

[72] Dominguez-Salas P Moore SE Baker MS Bergen AW Cox SE Dyer RA. Maternal nutrition at conception modulates DNA methylation of human metastable epialleles. Nat Commun. (2014) 5:3746. 10.1038/ncomms4746 24781383 PMC4015319

[73] Fleming TP Watkins AJ Velazquez MA Mathers JC Prentice AM Stephenson J. Origins of lifetime health around the time of conception: causes and consequences. Lancet. (2018) 391:1842–52. 10.1016/S0140-6736(18)30312-X 29673874 PMC5975952

[74] Malhotra A Elnakib S. 20 Years of the Evidence Base on What Works to Prevent Child Marriage: A Systematic Review. J Adolesc Health. (2021) 68:847–62. 10.1016/j.jadohealth.2020.11.017 33446401

[75] Brinda EM Rajkumar AP Enemark U. Association between gender inequality index and child mortality rates: a cross-national study of 138 countries. BMC Public Health. (2015) 15:97. 10.1186/s12889-015-1449-3 25886485 PMC4353466

[76] Marphatia AA Cole TJ Grijalva-Eternod CS Wells JC. Associations of gender inequality with child malnutrition and mortality across 96 countries. Global Health Epidemiol Genom. (2016) 1:e6. 10.1017/gheg.2016.1 29868199 PMC5870432

[77] Ewerling F Lynch JW Mittinty M Raj A Victora CG Coll CV. The impact of women's empowerment on their children's early development in 26 African countries. J Glob Health. (2020) 10:020406. 10.7189/jogh.10.020406 33214898 PMC7649042

[78] Chen L Li T King RB Du H Wu K Chi P. Gender Inequality lowers educational aspiration for adolescent boys and girls: a multi-level and longitudinal study in China. Sex Roles. (2022) 86:320–33. 10.1007/s11199-021-01272-z

[79] Prakash R Beattie TS Javalkar P Bhattacharjee P Ramanaik S Thalinja R. The Samata intervention to increase secondary school completion and reduce child marriage among adolescent girls: results from a cluster-randomised control trial in India. J Glob Health. (2019) 9:010430. 10.7189/jogh.09.010430 31448111 PMC6684866

[80] Adelman S Gilligan DO Konde-Lule J Alderman H. School feeding reduces anemia prevalence in adolescent girls and other vulnerable household members in a cluster randomized controlled trial in Uganda. J Nutr. (2019) 149:659–66. 10.1093/jn/nxy305 30926996 PMC6461720

