# Supplementary material for: Life history trade-offs associated with exposure to low maternal capital are different in sons compared to daughters: Evidence from a prospective Brazilian birth cohort
Source: Front Public Health. 2022 Sep 20;10:914965. doi: 10.3389/fpubh.2022.914965 (PMC9532015; doi:10.3389/fpubh.2022.914965)
Supplement: Supplementary file 1 [file Data_Sheet_1.PDF]

Supplementary online material for

Life history trade-offs associated with exposure to low maternal capital are different in sons compared to daughters: evidence from a prospective Brazilian birth cohort

Jonathan CK Wells, Tim J Cole, Mario Cortina-Borja, Rebecca Sear, David A Leon, Akanksha A Marphatia, Joseph Murray, Fernando César Wehrmeister, Paula Duarte Oliveira, Helen Gonçalves, Isabel O. Oliveira, Ana Maria Baptista Menezes

Missing data analysis

At 18 years, the proportion of missing data for son outcomes was as follows: 0.5% for reproductive status, education, work status and smoking status; 0.2% for birth weight; 0.8% for birth length; 1.3% for gestational age; 1.8% for blood pressure, 2.7% for anthropometry and body composition; 4.5% for blood biochemistry; and 6.8% for violent crime. We used *t*-tests to assess whether these data were missing at random in relation to maternal capital predictors.

For birth weight and birth length, there was no difference between those with or without maternal capital data, however mothers missing data on gestational age had greater education (1.9 years, 95%CI 0.3, 3.4) compared to those with data. For adult anthropometry and body composition, there were no differences between groups. For blood pressure and blood sampling, sons with missing maternal data had higher levels of education (BP: 1.7 years, 95%CI 0.5, 2.9; blood sampling: 1.0 years, 95%CI 0.1, 1.9) compared to those not missing data. Sons who lacked crime data had mothers who were older (1.5 years, 95%CI 0.4, 2.6), had higher family income (1.5 minimum wages, 95%CI 0.0, 3.0) and were less educated (1.0 years, 95%CI, 0.4, 1.6) compared to those with the data. No other differences were apparent in relation to maternal capital.

The proportion of missing data for maternal predictors was low, being 0% for parity, smoking status and age; 0.2% for education; 0.9% for height; 1.4% for income; 2.2% for weight; and 3.1% for BMI.

Supplementary Table 1. Differences between those followed up versus those not followed

	**Followed up (*n*=2024)**		**Not followed (*n*=579)**				
**Maternal characteristic**	*n*	%	*n*	%	OR*	95% CI	*p*-value
Parity 0 $	715	35	204	35	1.00	0.86, 1.17	0.9
Parity 4+ $	239	12	70	12	0.98	0.79, 1.22	0.8
Smoking in pregnancy $	649	32	194	34	0.99	0.82, 1.11	0.5
Alcohol in pregnancy $	90	4	33	6	1.22	0.90, 1.65	0.2
	Mean	SD	Mean	SD	Difference*	95% CI	*p*
Maternal age (y) #	25.6	6	26.1	7	0.4	-0.1, 1.0	0.14
Maternal height (cm) #	159.9	7	159.6	7	-0.2	-0.9, 0.4	0.4
Maternal BMI (kg/m^2) #	22.9	4	22.3	4	-0.5	-0.9, -0.2	0.004
Maternal education (y) #	6.8	4	6.7	4	-0.1	-0.4, 0.2	0.5
Family income (minimum wages) #	4.3	6	4.3	6	0.1	-0.5, 0.6	0.8
**Son characteristic**	Mean	SD	Mean	SD	Difference*	95% CI	*p*
Birth weight (g)	3251	534	3115	651	-135	-188, -27	<0.0001
Birth length (cm)	49.2	2.4	48.9	2.7	-0.3	-0.5, -0.0	0.023
Gestational age (weeks)	38.7	1.4	38.5	1.8	-0.2	-0.3, 0.0	0.029

* OR and difference values are calculated for those not followed up, relative to those followed up

$ Categorical outcomes compared by Chi-square test

Continuous outcomes tested by two-sample t-test

Supplementary Table 2A. Numeric results for comparisons of reproducing vs non-reproducing sons USING independent-samples t-tests

	**Data for Figure 2** Early-reproducing sons, relative to childless peers [1]	
Trait		
**Pregnancy traits**	**OR**	**95%CI**
Parity 0	0.74	0.51, 1.07
Parity 4+	1.90	1.23, 2.92
Smoking in pregnancy	1.70	1.21, 2.38
Alcohol in pregnancy	0.71	0.35, 1.45
**Developmental traits**	**% difference**	**95%CI**
Birth weight (g)	-2.2	-5.1, 0.7
Birth length (cm)	-0.2	-1.1, 0.6
Gestational age (weeks)	-0.5	-1.2, 0.1
Weight 1 year (kg)	-2.3	-6.3, 1.7
Length 1 year (cm)	-0.7	-2.0, 0.6
**Adult physical traits**	**% difference**	**95%CI**
Height (cm)	-1.4	-2.1, -0.7
Weight (kg)	-3.6	-6.8, -0.4
BMI (kg/m^2)	-0.8	-3.6, 2.0
Fat-free mass (kg)	-1.2	-3.3, 0.9
Fat mass (kg)	-15.0	-26.8, -3.1
Fat-free mass index (kg/m^2)	1.6	0.0, 3.3
Fat mass index (kg/m^2)	-12.2	-23.8, -0.5
Triceps skinfold (mm)	-11.5	-20.6, -2.4
Subscapular (mm)	-3.0	-9.3, 3.4
Glucose (mg/dL)	-2.3	-5.3, 0.7
Cholesterol (mg/dL)	-2.3	-5.0, 0.4
HDL (mg/dL)	-1.7	-4.6, 1.1
LDL (mg/dL)	-2.8	-6.8, 1.3
Triglycerides (mg/dL)	-4.4	-11.3, 2.6
HbA1c (%)	-1.5	-3.4, 0.4

Systolic BP (mmHG)	-1.8	-3.3, -0.3
Diastolic BP (mmHG)	-1.2	-3.1, 0.6
	**OR**	**95%CI**
Studying past year	0.19	0.13, 0.27
Studying now	0.17	0.11, 0.26
Received allowance in last month	2.04	1.35, 3.07
Received payment for work in last year	3.63	1.32, 9.95
Smoked at least once in last week	3.61	2.60, 5.06
Current smoker	3.56	2.49, 5.10
Violent crime	2.55	1.75, 3.72

[1] Coefficients for early-reproducing sons (n=150) relative to non-reproducing sons (*n*=1864)

Categorical variables assessed by chi-square test (expressed as odds ratio (OR) and 95% confidence intervals (CI)

Continuous variables assessed after natural log transformation by independent-samples t-test (multiplied by 100%, expressed as % difference) and 95% confidence intervals (CI)

Supplementary Table 2. Numeric results for comparisons of reproducing vs non-reproducing sons USING Mann-Whitney U-tests

	**Data for Figure 2** Early-reproducing sons, relative to childless peers [1]	
Trait		
**Pregnancy traits**	**OR**	**95%CI**
Parity 0	0.74	0.51, 1.07
Parity 4^{+}	1.90	1.23, 2.92
Smoking in pregnancy	1.70	1.21, 2.38
Alcohol in pregnancy	0.71	0.35, 1.45
**Developmental traits**	**% difference**	**95%CI**
Birth weight (g)	-1.4	-4.2, -1.3
Birth length (cm)	0.0	-1.0, 0.0
Gestational age (weeks)	-0.5	-1.2, 0.1
Weight 1 year (kg)	-2.3	-6.3, 1.7
Length 1 year (cm)	-0.7	-2.0, 0.6
**Adult physical traits**	**% difference**	**95%CI**
Height (cm)	-1.4	-2.8, -0.0
Weight (kg)	-3.1	-6.1, -0.1
BMI (kg/m^2)	-0.0	-2.5, 2.5
Fat-free mass (kg)	-1.0	-3.0, 1.0
Fat mass (kg)	-16.4	-28.1, -4.5
Fat-free mass index (kg/m^2)	1.8	0.2, 3.4
Fat mass index (kg/m^2)	-13.6	-25.2, -2.0
Triceps skinfold (mm)	-12.3	-20.9, -3.6
Subscapular (mm)	-1.9	-7.2, 3.4
Glucose (mg/dL)	-1.3	-4.4, 1.1
Cholesterol (mg/dL)	-1.9	-4.2, 0.6
HDL (mg/dL)	-2.0	-5.0, 0.0
LDL (mg/dL)	-1.4	-5.2, 1.5
Triglycerides (mg/dL)	-5.5	-11.4, 0.0
HbA1c (%)	-1.9	-3.8, 0.0

Systolic BP (mmHG)	-2.1	-3.6, -0.8
Diastolic BP (mmHG)	-0.8	-2.9, 0.7
	**OR**	**95%CI**
Studying past year	0.19	0.13, 0.27
Studying now	0.17	0.11, 0.26
Received allowance in last month	2.04	1.35, 3.07
Received payment for work in last year	3.63	1.32, 9.95
Smoked at least once in last week	3.61	2.60, 5.06
Current smoker	3.56	2.49, 5.10
Violent crime	2.55	1.75, 3.72

[1] Coefficients for early-reproducing sons (n=150) relative to non-reproducing sons (n=1864)
Categorical variables assessed by chi-square test (expressed as odds ratio (OR) and 95% confidence intervals (CI)
Continuous variables assessed after natural log transformation by independent-samples t-test (multiplied by 100%, expressed as % difference) and 95% confidence intervals (CI)

Supplementary Table 3. Dose-response associations of individual maternal capital components according to the number of penalties in the composite maternal capital index

			Number of maternal capital penalties relative to high maternal capital (0 penalties)								
	High maternal capital		1		2		3		4		*p*-value
	Coeff	95%CI	Coeff	95%CI	Coeff	95%CI	Coeff	95%CI	Coeff	95%CI	
Maternal height (cm)	163.6	162.9, 164.2	-2.0	-2.7 -1.2	-3.8	-4.6, -3.0	-7.4	-8.3, -6.5	-11.3	-12.7, -9.8	<0.0001
Maternal BMI (kg/m^2)	24.6	24.2, 25.0	-1.6	-2.1, -1.1	-1.8	-2.4, -1.3	-2.9	-3.4, -2.3	-5.1	-6.0, -4.2	<0.0001
Maternal income (wages)	8.1	7.5, 8.7	-2.2	-2.9, -1.4	-5.4	-6.2, -4.7	-6.3	-7.1, -5.4	-6.6	-7.9, -5.4	<0.0001
Maternal education (years)	10.2	9.9, 10.5	-1.9	-2.2, -1.5	-4.4	-4.8, -4.0	-6.0	-6.4, -5.6	-6.7	-7.3, -6.0	<0.0001

p-value for trend

[$] coefficients for groups with1 to 4 penalties in maternal capital (short stature, low BMI, low education and low income) relative to group with no penalties

Supplementary Table 4. Dose response associations of sons' cardio-metabolic traits according to the number of penalties in maternal capital, adjusting for sons' fat mass

Predictor	0 penalties (*n*=389)		1 penalty (*n*=617)		2 penalties (*n*=573)		3 penalties (*n*=299)		4 penalties (*n*=69)		
	Constant		1 penalty[$]		2 penalties[$]		3 penalties[$]		4 penalties[$]		*p*-value[2]
**Son Traits**	Coeff	95%CI	Coeff	95%CI	Coeff	95%CI	Coeff	95%CI	Coeff	95%CI	
Glucose (mg/dL)	89.8	86.6, 93.0	1.8	-1.4, 5.0	1.4	-1.8, 4.6	2.1	-1.5, 5.7	3.7	-1.9, 9.4	0.2
HbA1c (%)	4.99	4.91, 5.07	0.02	-0.05, 0.10	-0.00	-0.08, 0.08	-0.04	-0.13, 0.04	-0.06	-0.20, 0.07	0.1
Cholesterol (mg/dL)	146.7	143.5, 149.8	-3.0	-6.2, 0.2	-4.7	-7.9, -1.4	-5.1	-8.7, -1.5	-4.1	-9.8, 1.5	0.0007
HDL (mg/dL)	55.0	53.8, 56.2	-1.3	-2.5, -0.1	-1.9	-3.1, -0.7	-2.2	-3.5, -0.8	-4.5	-6.7, -2.4	<0.0001
LDL (mg/dL)	77.2	74.6, 79.8	-1.3	-4.0, 1.3	-1.9	-4.6, 0.8	-2.5	-5.5, 0.4	-0.0	-4.6, 4.7	0.2
HDL/Cholesterol ratio	0.37	0.37, 0.38	-0.00	-0.01, 0.01	-.00	-0.01, 0.01	-.00	-0.01, 0.01	-0.02	-0.03, 0.01	0.059
Triglycerides (mg/dL)	65.7	58.4, 72.9	-3.8	-11.1, 3.5	-6.5	-13.9, 0.9	-6.2	-14.4, 2.0	-3.0	-16.0, 9.9	0.17
Systolic BP (mmHg)	127.9	126.4, 129.5	-1.0	-2.6, 0.6	-0.1	-1.7, 1.5	-2.1	-3.9, -0.3	-2.6	-5.5, 0.2	0.033
Diastolic BP (mmHg)	67.4	66.4, 68.4	0.0	-1.0, 1.1	1.0	0.1, 2.1	-0.7	-1.2, 1.1	0.2	-1.7, 2.1	0.5

[$]Each outcome was regressed on four dummy variables, whereby the son's mother was identified as having 1, 2 3 or 4 capital penalties (0 penalties = reference group)
The mean coefficient and its 95% CI intervals are shown for each dummy variable. Penalties refer to maternal short stature, low BMI< low education or low family income.
[¥] HbA1c shown as percentage of total haemoglobin
n=2024, small level of missing data for body composition and cardio-metabolic outcomes as described in text

Supplementary Table 5. Odds ratio of early reproduction associated with son's growth phenotype, adjusted for maternal capital

Predictor	Odds Ratio	95% CI
Weight 18 y (kg)	0.99	0.98, 1.01
Maternal capital penalty 1	2.06	0.97, 4.38
Maternal capital penalty 2	2.28	1.07, 4.84
Maternal capital penalty 3	4.90	2.32, 10.37
Maternal capital penalty 4	6.47	2.62, 15.98
Height 18 y (kg)	0.97	0.94, 0.99
Maternal capital penalty 1	1.98	0.93, 4.19
Maternal capital penalty 2	2.14	1.01, 4.53
Maternal capital penalty 3	4.30	2.04, 9.07
Maternal capital penalty 4	5.27	2.13, 13.07
Fat mass index 18 y (kg/m^2)	0.96	0.90, 1.03
Maternal capital penalty 1	2.06	0.97, 4.35
Maternal capital penalty 2	2.27	1.08, 4.80
Maternal capital penalty 3	4.91	2.35, 10.26
Maternal capital penalty 4	6.65	2.67, 15.74
Fat-free mass index 18 y (kg/m^2)	1.11	1.01, 1.22
Maternal capital penalty 1	2.24	1.06, 4.75
Maternal capital penalty 2	2.55	1.21, 5.38
Maternal capital penalty 3	5.74	2.75, 11.99
Maternal capital penalty 4	7.79	3.22, 18.87

Logistic regression analyses

Supplementary Table 6. Odds ratio of early reproduction associated with son's behaviour, adjusted for maternal capital

Predictor	Odds Ratio	95% CI
Not studied last year	0.23	0.16, 0.32
Maternal capital penalty 1	1.81	0.85, 3.85
Maternal capital penalty 2	1.66	0.78, 3.54
Maternal capital penalty 3	3.62	1.72, 7.63
Maternal capital penalty 4	4.53	1.83, 11.17
Smoking	3.37	2.36, 4.81
Maternal capital penalty 1	2.10	0.99, 4.44
Maternal capital penalty 2	1.97	0.93, 4.18
Maternal capital penalty 3	4.75	2.28, 9.93
Maternal capital penalty 4	5.43	2.22, 13.25
Violent crime	2.52	1.71, 3.73
Maternal capital penalty 1	2.70	1.18, 6.21
Maternal capital penalty 2	2.37	1.02, 5.50
Maternal capital penalty 3	5.83	2.56, 13.28
Maternal capital penalty 4	7.28	2.64, 20.08

Logistic regression analyses
